# Ocean currents magnify upwelling and deliver nutritional subsidies to reef-building corals during El Niño heatwaves

**DOI:** 10.1126/sciadv.add5032

**Published:** 2023-06-14

**Authors:** Michael D. Fox, Robin Guillaume-Castel, Clinton B. Edwards, J. Glanz, Jamison M. Gove, J. A. Mattias Green, E. Juhlin, Jennifer E. Smith, Gareth J. Williams

**Affiliations:** ^1^ Division of Biological and Environmental Science and Engineering, King Abdullah University of Science and Technology (KAUST), Thuwal, Saudi Arabia.; ^2^ Marine Science Program, KAUST, Thuwal, Saudi Arabia.; ^3^ Red Sea Research Center, KAUST, Thuwal, Saudi Arabia.; ^4^ LEGOS, Université de Toulouse, CNES, CNRS, Toulouse, France.; ^5^ School of Ocean Sciences, Bangor University, Anglesey LL59 5AB, UK.; ^6^ Center for Marine Biodiversity and Conservation, Scripps Institution of Oceanography, University of California San Diego, La Jolla, CA, USA.; ^7^ National Oceanic and Atmospheric Administration, Pacific Islands Fisheries Science Center, Honolulu, HI, USA.

## Abstract

Marine heatwaves are triggering coral bleaching events and devastating coral populations globally, highlighting the need to identify processes promoting coral survival. Here, we show that acceleration of a major ocean current and shallowing of the surface mixed layer enhanced localized upwelling on a central Pacific coral reef during the three strongest El Niño–associated marine heatwaves of the past half century. These conditions mitigated regional declines in primary production and bolstered local supply of nutritional resources to corals during a bleaching event. The reefs subsequently suffered limited post-bleaching coral mortality. Our results reveal how large-scale ocean-climate interactions affect reef ecosystems thousands of kilometers away and provide a valuable framework for identifying reefs that may benefit from such biophysical linkages during future bleaching events.

## Teaser

Ocean currents enhance upwelling and promote coral survival during El Niño heatwaves.

## INTRODUCTION

Oceanographic processes that originate beyond the coastal margins of coral reefs strongly influence the physical, chemical, and biological conditions of these dynamic ecosystems (*1*). Marine heatwaves are intensifying coral bleaching events globally (*2*), raising important questions about how heatwaves modify oceanographic processes, such as upwelling, that supply crucial nutritional resources to corals (*3*, *4*). In some locations, upwelled nutrients may exacerbate coral sensitivity to thermal stress (*5*), while, in others, upwelling can provide thermal refuge (*6*, *7*) or deliver food subsidies that might facilitate coral survival during bleaching (*8*, *9*). Determining how heatwaves modify key oceanographic processes is, therefore, critical for resolving variability in coral reef trajectories under ocean warming.

Oceanic primary production fuels pelagic subsidies to coral reef food webs (*10*–*12*) but is expected to decline under future ocean warming (*13*) with unknown consequences for reefs in oligotrophic regions. The central Pacific provides an opportunity to explore the impact of declining pelagic subsidies, primarily nutrient concentrations and planktonic biomass, to coral reefs during heat waves because sea surface temperature and primary production are inversely related during strong El Niños (*14*). The 2015–2016 El Niño generated unprecedented thermal stress and reduced surface chlorophyll *a* concentrations across the central Pacific, with severe consequences for coral, fish, and seabird populations (*15*, *16*). The loss of pelagic subsidies to reefs during heatwaves might exacerbate the impacts of thermal stress or delay recovery by removing a critical source of heterotrophic nutrition for bleached corals, which lose their primary source of energy after expelling their algal endosymbionts (*9*). However, nutritional resources are also supplied to corals by localized processes including upwelling and lagoonal flushing (*1*, *3*, *17*). These island-scale processes may be less affected by heatwaves than regional processes, yet their potential importance as pathways of resource supply during bleaching events remains unknown.

Here, we show that regional strengthening of a major ocean current and shoaling of the surface mixed layer can enhance localized upwelling and food supply to coral reefs during strong El Niños. We quantified temporal changes in coral trophic ecology for 2 years before and during the 2015–2016 El Niño bleaching event and integrated these observations with macroalgal δ^15^N data, subsurface temperature measurements, and 40 years of regional oceanographic data to identify the physical drivers governing nutritional subsidies to a central Pacific coral reef. Our findings reveal that increased ocean current speed and a shallow surface mixed layer can locally enhance upwelling in the central Pacific, delivering nutritional subsidies to off-equatorial coral populations during bleaching. We further show that these coupled oceanographic processes are a recurrent feature in this region, occurring during all major El Niño event over the past half century. These findings reveal a previously unknown mechanism of upwelling enhancement that can subsidize coral reefs during heatwaves and regional desertification. Mechanisms of resource supply, such as the one identified here, likely benefit reefs worldwide. Resolving the diversity of these mechanisms and their influence on coral survival and recovery will provide a valuable framework for refining spatial and temporal projections of coral reef vulnerability to climate change.

## RESULTS AND DISCUSSION

The influence of physical drivers on coral reef dynamics is challenging to isolate on populated reefs. Local human impacts can overwhelm natural processes that help structure reefs (*18*) and erode their ability to cope with heatwaves (*19*). By contrast, uninhabited islands provide an opportunity to more accurately resolve physical processes that can modulate coral survival and recovery from bleaching. Palmyra (5.89°N 162.08°W) is a federally protected, uninhabited coral reef atoll in the central Pacific Ocean with a stable, well-documented coral reef community (*20*–*23*). Localized upwelling provides nutritional resources to Palmyra’s reef community (*17*), making this the ideal location to determine how upwelling and resource supply were altered during the 2015–2016 El Niño–associated bleaching event (*24*).

### Corals exhibit reduced trophic diversity and increased heterotrophy during El Niño

**Fig. 1.**
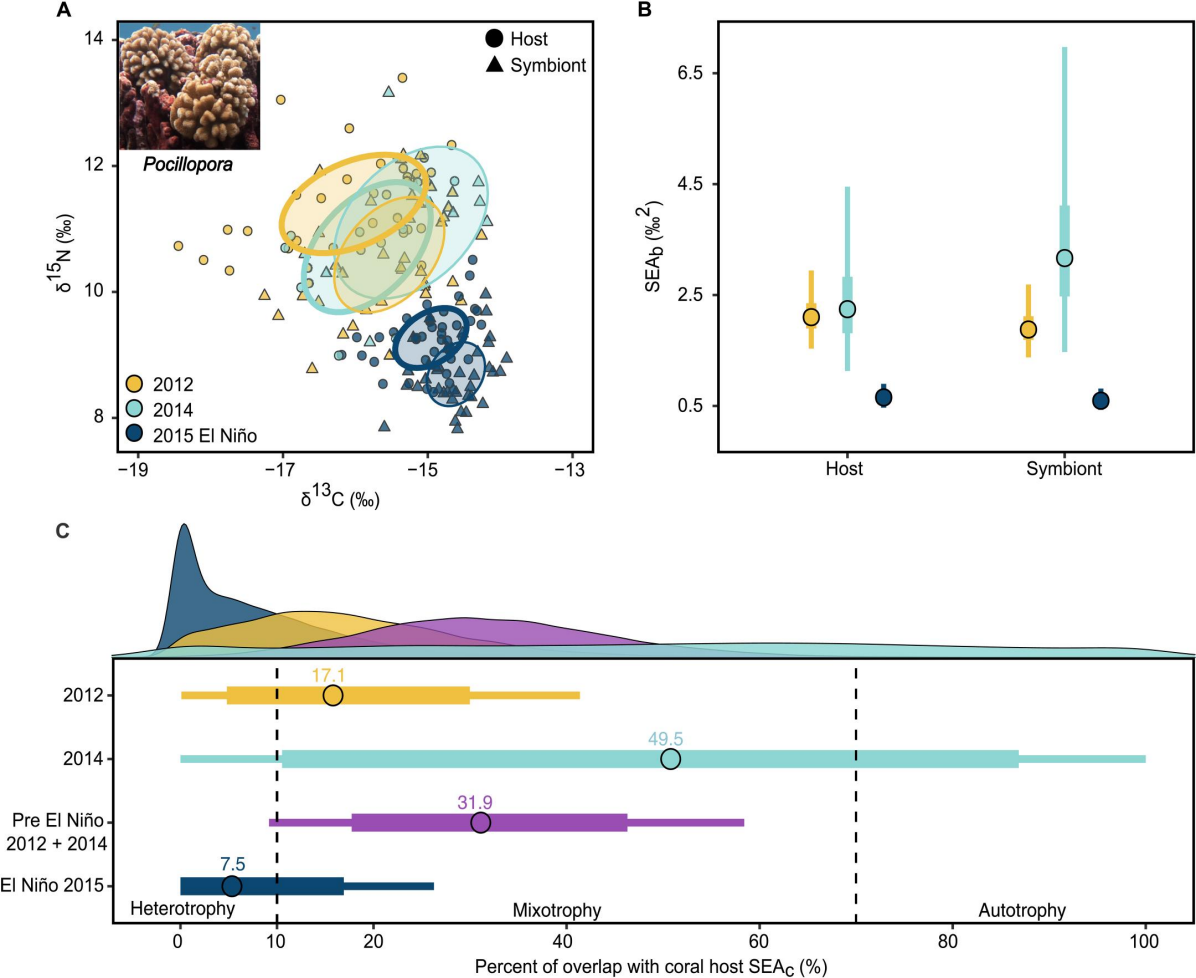
Interannual variation in *Pocillopora meandrina* stable isotope values reveals isotopic niche compression and a shift toward heterotrophy during the 2015 El Niño. (**A**) Standard ellipse areas corrected for sample size (SEA_c_) for coral host (circles, thick ellipse) and endosymbionts (triangle, thin ellipse). Less overlap between host and symbiont SEA_c_ indicates greater contribution of heterotrophy. Permutational tests of the Euclidean distance and Hollings *T*^2^ test between the host and symbiont niches confirm that they occupy distinct isotopic space in 2012 and 2015 (not 2014). (**B**) Bayesian standard ellipse areas (SEA_b_) estimates reveal substantial compression of isotopic niches during El Niño. Points represent the mode with 50% and 95% credible intervals. (**C**) Bootstrapped estimates of SEA_c_ overlap as a proxy for coral trophic strategy. Overlap between the coral and symbiont niches is quantified as the percentage of coral host SEA_c_ overlapping symbiont SEA_c_. The distributions of 10,000 overlap estimates are shown along the top margin. Mean overlap ± 75% (thick) and 95% confidence intervals (thin) are shown for each year (sample sizes for 2012, *n* = 38; 2014, *n* = 10; and 2015, *n* = 39). To reflect natural variation in the *Pocillopora* isotopic niche outside of El Niño, data from 2012 and 2014 were pooled to estimate the average SEA_c_ overlap (purple). The distinction of coral trophic strategies (dashed lines) is based on the % overlap cutoffs established by (*26*). See fig. S2 to S4 for interannual and spatial information about coral and endosymbiont δ^13^C and δ^15^N values and tables S1 to S3 for statistical analysis of coral trophic niches.

We sampled the reef-building coral, *Pocillopora meandrina*, from four locations that span the east-west and north-south exposures of Palmyra’s outer reef slope (10 m depth; fig. S1A) in September 2012, 2014, and 2015 to examine interannual variation in coral trophic ecology. We used Bayesian analysis of isotopic niches (*25*) and the six Layman metrics of trophic diversity (*24*) to quantify changes in the isotopic niche area of *P. meandrina*. In 2012 and 2014, the *P. meandrina* community exhibited similarly broad isotopic niche areas (Fig. 1A) associated with spatial heterogeneity in trophic strategy governed by gradients in upwelling and lagoonal flushing (*17*). By contrast, the isotopic niche area of both the coral host and endosymbionts was markedly compressed during the El Niño in 2015. Bayesian standard ellipse area (SEA_b_) was reduced by ~70% relative to non–El Niño years (Fig. 1B), and all Layman metrics decreased by 35 to 74% (table S2), indicating lower isotopic and trophic diversity of *P. meandrina* across the Atoll. This compression was driven by a 2 per mil (‰) decline in average δ^15^N values and >40% reduction in the observed δ^13^C range in the coral host and endosymbionts (Fig. 1A, figs. S2 to S4, and tables S1 and S2). The reduced isotopic diversity and declines in δ^15^N values during the El Niño imply a spatial homogenization of coral δ^13^C and δ^15^N values and a fundamental shift in the baseline nitrogen source across Palmyra relative to non–El Niño years (figs. S2 to S4).

We quantified the relative reliance of heterotrophy versus autotrophy in *P. meandrina* as the percentage overlap between coral and endosymbiont standard ellipse areas corrected for sample size (SEA_c_) (*26*). Using bootstrap resampling, we conservatively estimated SEA_c_ overlap to account for overestimates of heterotrophy caused by changes in the δ^13^C and δ^15^N baselines between years and the stark reduction in isotopic variation during 2015 (*27*). Because of a smaller sample size in 2014 and consistent overlap with isotopic niches in 2012 (Fig. 1A), we pooled data for these years to estimate the *P. meandrina* isotopic niche area during non–El Niño conditions. During El Niño, there was a 77% reduction in SEA_c_ overlap relative to this baseline, indicating increased heterotrophy by *P. meandrina* (Fig. 1C and table S3). Mixotrophic corals can increase heterotrophy when nutritional resources are abundant (*11*, *28*–*30*), setting up the expectation that heterotrophy should have declined in parallel with the reductions in regional primary production, or food availability, associated with El Niño (*15*). By contrast, increased heterotrophy by *P. meandrina* suggests food availability was locally maintained, enabling access to nutritional resources during bleaching. No sampled colonies showed evidence of paling or bleaching at the time of collection, which was before the onset of widespread bleaching at Palmyra. It is, therefore, unlikely this increase in heterotrophy was forced by a loss of endosymbionts and reduction in autotrophic nutrition. It is possible, however, that increased energetic demands associated with heat stress in *P. meandrina* magnified the observed increase in heterotrophy. Resource availability during periods of thermal stress likely has species-specific benefits that will favor corals with the most flexible trophic strategies or those that naturally rely on a higher degree of heterotrophic nutrition (*26*). Broader taxonomic evaluations of coral trophic strategies will, therefore, improve projections of changes in coral community composition under climate change.

**Fig. 2.**
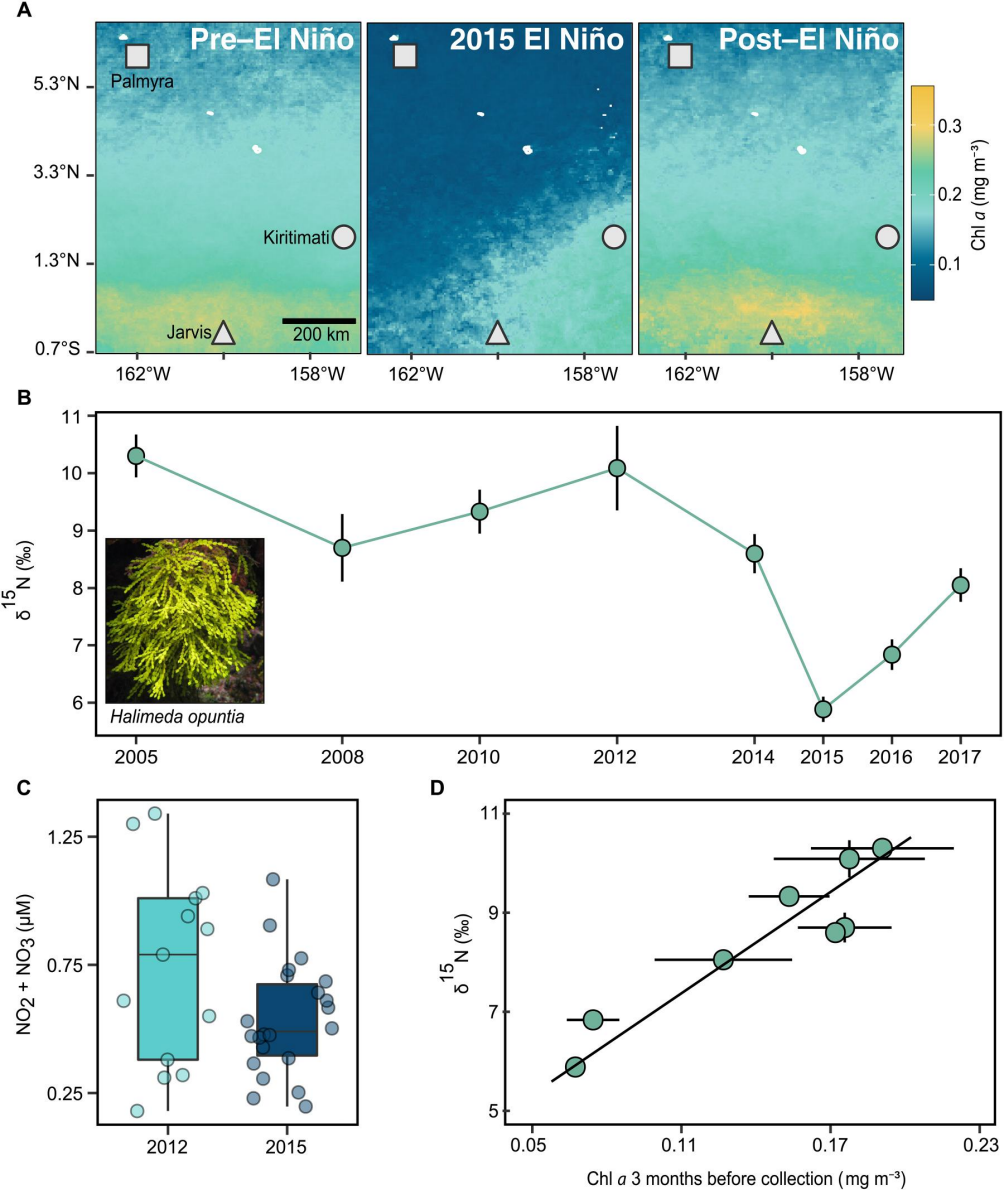
Regional variations in surface ocean chlorophyll *a* concentrations are captured in the δ^15^N values of the pantropical reef alga, *H. opuntia*. (**A**) Mean surface chlorophyll *a* across the Northern Line Islands between May and September. Pre–El Niño includes all years in (**B**) before 2015–2016, and post–El Niño is 2017. (B) Annual mean *Halimeda* *opuntia *δ^15^N ± 95% CI from 10 m depth on the outer reef slope of Palmyra Atoll during September (2010 collected in early October). (**C**) In situ measurements of dissolved inorganic nitrogen (DIN = NO_2_ + NO_3_) collected from the outer reef slope in 2012 (*n* = 4 sites, 13 replicates) and 2015 (*n* = 13 sites, 22 replicates). (**D**) Mean *Halimeda* δ^15^N values are most strongly related to nearshore chlorophyll *a* (chl *a*) values near Palmyra with a 3-month lag. Vertical error bars ± 95% CI (small error bars may be obscured) and horizontal bars ±1 SD. The line represents best fit of standard major axis regression (*R*^2^ = 0.86). See fig. S5 for additional *Halimeda* tissue chemistry data and tables S4 and S5 for statistical summaries for (B) and (D).

Satellite-derived estimates of surface ocean chlorophyll *a* concentrations in the central Pacific are a reliable proxy for phytoplankton biomass (*1*) and zooplankton abundance (*31*). At Palmyra, surface chlorophyll *a* concentrations declined by 56% during the 2015–2016 El Niño relative to long-term baseline conditions (Fig. 2A). The expectation is that this desertification reduces pelagic resource supply, resulting in decreased levels of planktonic predation by corals (*11*). The discrepancy between decreased regional primary production and elevated coral heterotrophy suggests that more localized and subsurface processes may supply nutritional resources. Localized upwelling supplies food to corals on Palmyra’s outer reef slope, but its influence is geographically constrained and upwelled waters do not always reach the surface (*17*), exceeding the detection capabilities of most space-based ocean sensors.

### Macroalgal collections reveal a shift in nitrogen supply during El Niño

An 8-year times series of δ^15^N values in a pantropical reef macroalga, *Halimeda opuntia*, (hereafter *Halimeda*) collected at 10 m depth from our sampling sites revealed a substantial shift in the dominant nitrogen source on Palmyra during the 2015–2016 El Niño (Fig. 2B). The δ^15^N values of macroalgae reliably track nitrogen inputs from upwelling (*32*), and, on Palmyra, macroalgal δ^15^N values distinguish lagoon versus pelagic nitrogen sources (*17*). The pre–El Niño (2005–2014) *Halimeda* δ^15^N baseline ranged from 8.6 to 10.3‰ (mean, 9.4‰ ± 0.8 SD) (Fig. 2B and fig. S5) but dropped precipitously to 5.9‰ ± 0.5 during El Niño. *Halimeda* carbon-to-nitrogen (C:N) ratios increased rather than decreased during 2015, implying that the change in dominant nitrogen source did not result in substantially elevated nutrient concentrations on the reef at our sampling depth (fig. S5 and table S4). This is further supported by in situ measurements of dissolved inorganic nitrogen (DIN) at 10 m depth at our four sampling sites in 2012 (*n* = 13, mean = 0.74 μmol ± 0.38 SD) and at 13 sites spanning the circumference of Palmyra’s outer reef slope in 2015 (*n* = 22, mean = 0.54 ± 0.02 SD) (Fig. 2C). While capturing a cold pulse during in situ water sampling is improbable, these results suggest that the increased upwelling did not appreciably increase the background mean DIN concentrations at 10 m depth during our sampling in September 2015.

This distinct nitrogen source was pervasive across Palmyra in 2015, where it was incorporated by reef primary producers and propagated through the food web. Mean *P. meandrina* host tissue δ^15^N (9.3‰ ± 0.5) was reduced by 2‰ relative to non–El Niño years but remained 3 to 4‰ above the primary producer baseline (*Halimeda* δ^15^N = 5.9‰ ± 0.5), consistent with theory that predicts an enrichment between successive trophic levels (Figs. 1 and 2B and fig. S2 to S5) (*26*). The similar declines of coral and endosymbiont δ^15^N values, invariant C:N ratios of the endosymbionts, and the low DIN concentrations measured at our collection sites further support that observed changes in the coral δ^15^N values were not due to increased nitrogen uptake by the endosymbionts alone (fig. S2 and table S1). Under elevated nutrient concentrations and without substantial heterotrophy, the δ^15^N value of *P. meandrina* would likely have remained similar to non–El Niño years while endosymbiont C:N ratios would have strongly declined, resulting in δ^15^N values more similar to *Halimeda* (*33*).

Between 2005 and 2017, *Halimeda* δ^15^N values were greatest during periods of high surface chlorophyll *a* concentrations around Palmyra and lowest during the regional desertification associated with El Niño (*15*). Monthly mean surface chlorophyll *a* values around Palmyra are highest in May and June. δ^15^N values of *Halimeda* strongly track average chlorophyll *a* concentrations with a three-month lag (*R*^2^ = 0.86, *P* < 0.001; Fig. 2D and table S5), reaffirming a tight linkage between large-scale regional primary production and reef-associated organisms. Equatorial upwelling is the dominant source of nitrogen in the central Pacific in non–El Niño years, but it is depressed during El Niño (*14*) when the equatorial undercurrent (EUC) stops flowing (*34*). There was no evidence for locally elevated N_2_ fixation (δ^15^N = −1‰) in Palmyra’s lagoon that might help explain reduced δ^15^N values (see Supplementary Materials and Methods). Rather, our results are consistent with the hypothesis that the cessation of equatorial upwelling occurred alongside a regional decline in surface ocean primary production (Fig. 2A) during the 2015 El Niño. This likely resulted in less partial utilization of the nitrogen pool, reduced Raleigh Fractionation across the central Pacific (*35*), and a more localized nitrogen source for Palmyra (nitrate δ^15^N = ~7‰) (*36*). Nevertheless, contributions of N_2_ fixation in the North Pacific Gyre or eastern Pacific cannot be ruled out (*37*), and these findings should catalyze further investigation to Pacific-wide nutrient transport during El Niño. Our observations provide a salient example of interconnected ocean processes, where biophysical processes in one location can influence local-scale resource supply and ecosystem response thousands of kilometers away.

### Localized upwelling enhances resource supply during El Niño

**Fig. 3.**
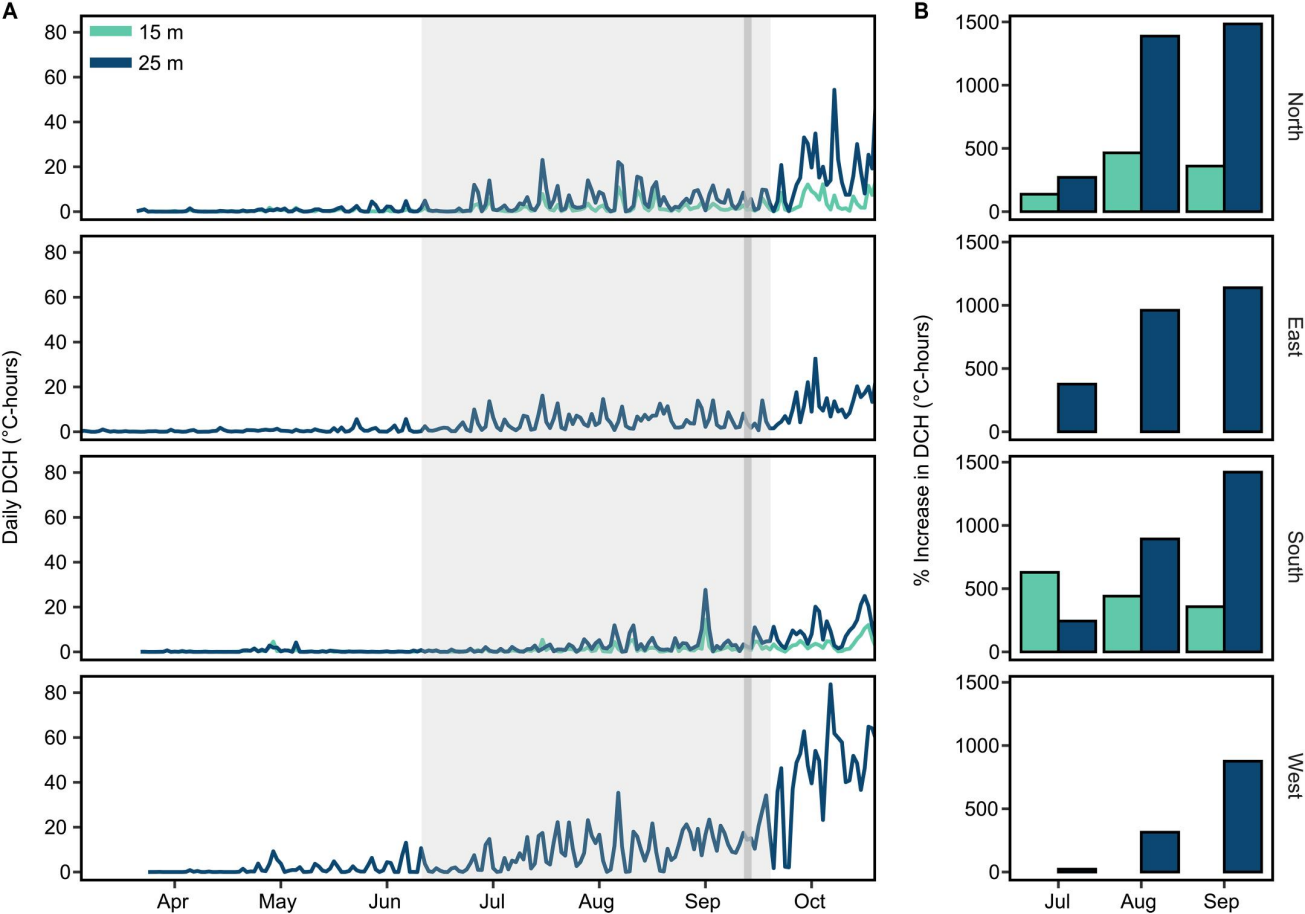
Subsurface in situ temperature measurements reveal island-wide upwelling enhancement on Palmyra during El Niño. (**A**) Daily degree cooling hours (DCHs) at 15 and 25 m depth from April to October 2015. DCH provides an integrated upwelling metric that accounts for both the magnitude and duration of cold pulses such that a cold pulse that reduces temperature by 2°C and lasts for 2 hour is equal to 4 DCHs. The shaded region encompasses July to September, the period used to calculate upwelling enhancement in (**B**). Narrow gray bars depict 24 to 26 September, when biological samples were collected. No DCH data are available at 15 m on the east and west because there was no temperature sensor shallower than 15 m. (B) The % increase in total DCH per month relative to the average monthly DCH for all years between 2012 and 2017 (excluding 2015). The % increase for the west is relative to DCH in 2016 and 2017 as there is no pre–El Niño data from this location. Enhancement in DCH at 15 m depth, closest to our sampling depth, ranged from 138 to 465% above average in the north and 357 to 629% in the south for the 3 months before sampling. The proportional increase is lowest in the west because background upwelling is greatest on this side of the Atoll during non–El Niño conditions. See fig. S6 for data between 1980 and 2020 and tables S6 and S7 for metadata from NOAA temperature loggers used to quantify cold pulses.

High-frequency upwelling events on Palmyra influence the trophic ecology of mixotrophic corals (*17*). As such, we measured subsurface in situ temperature at multiple locations on Palmyra’s outer reef slope (5 m, 15 and 25 m depth; fig. S1A and tables S5) between 2009 and 2017 to determine how upwelling was affected by the 2015–2016 El Niño. To contextualize changes in upwelling during 2015, we focused on four locations with data between 2012 and 2017 that represent the north, south, east, and west extremes of Palmyra’s outer reef slope (fig. S1A and table S6). We used a temperature stratification index (TSI) to detect individual pulses of cold water propagating up the reef slope (*38*) and standardized the magnitude and duration of these “cold pulses” during each day as cumulative degree cooling hours (DCHs). Upwelling increased markedly at all sites during El Niño, peaking between September and November 2015 (Fig. 3A), coincident with peak thermal stress on the coral community (*39*). Mean monthly DCH increased 398% ±162 SD at 15 m depth and 782% ± 518 at 25 m depth, with a >1200% increase on the northern coast at 25 m depth in September in 2015 relative to non–El Niño Septembers (Fig. 3B). The surge in local upwelling, despite regional desertification, likely supplied the reef communities with nutritional resources for at least 3 months before our 2015 sampling. During non–El Niño years, upwelling is strongest on Palmyra’s western coast where the eastward flowing North Equatorial Counter Current (NECC) first makes physical contact (*17*, *21*). However, in 2015, upwelling was enhanced around the entire atoll, homogenizing intra-island gradients in energetic resources and subsidizing parts of the reef less commonly exposed to upwelling.

**Fig. 4.**
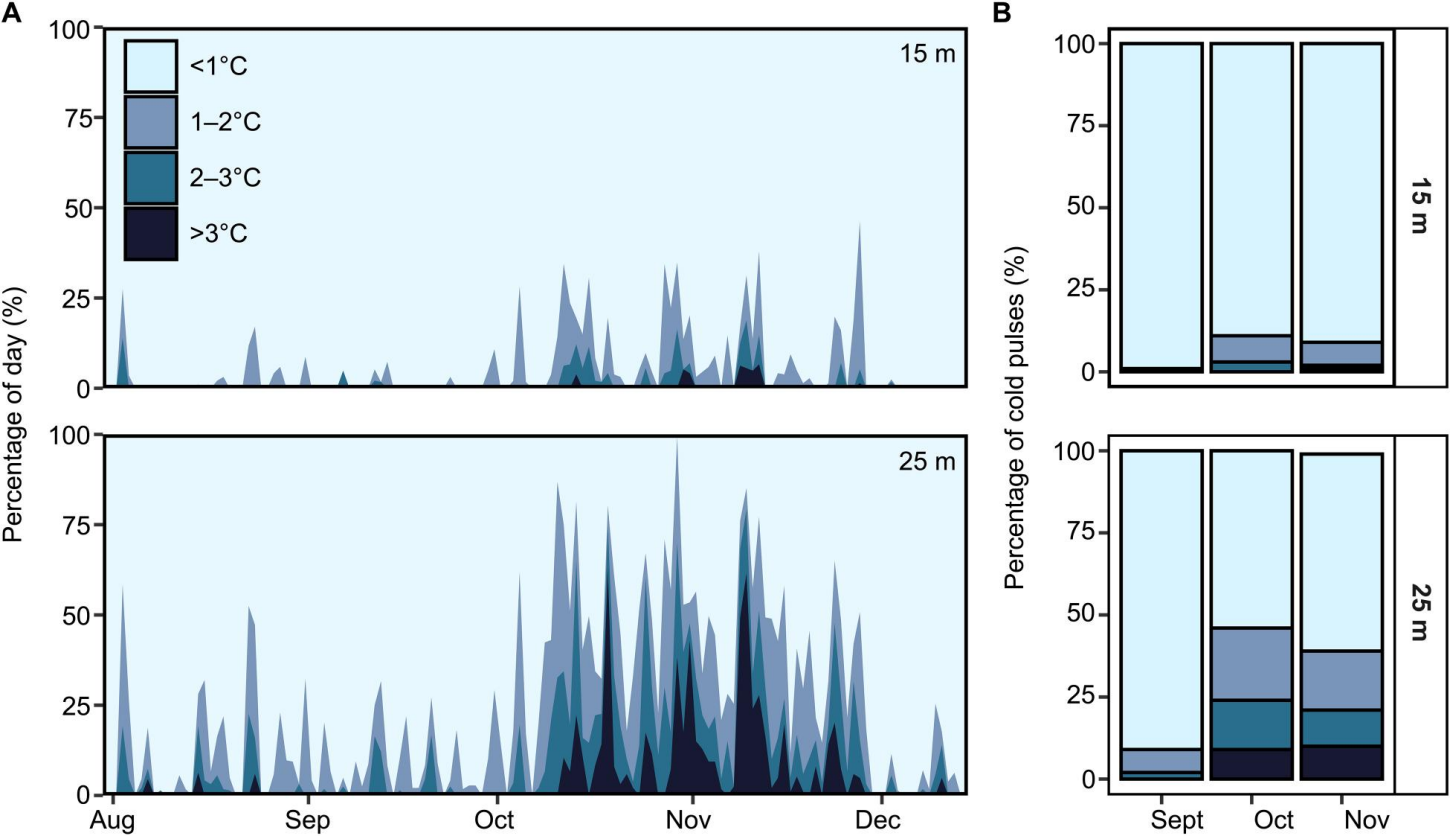
The cooling effect of cold pulses on the outer reef slope at Palmyra is strongly depth restricted. (**A**) The daily percentage of time influenced by cold pulses of varying magnitudes (degrees below ambient). The light blue background denotes periods of time where no cold pulses are present or cold pulses that reduced temperature by less than 1°C. The darker blue areas indicate the magnitude and duration of all pulses that resulted in temperature declines of greater than 1°C. (**B**) The total percentage of cold pulses of different magnitudes that occurred during the 3 months with the greatest upwelling activity. See fig. S1 for in situ temperature data at 10 m depth illustrating cold pulses detected at the depth of coral sampling and tables S6 and S7 for metadata from NOAA temperature loggers used to quantify cold pulses.

We confirmed the influence of cold pulses at 10 m depth with in situ temperature measurements from our sampling site on Palmyra’s north shore. While unable to resolve individual cold pulses, these data captured distinct, high-frequency temperature drops, consistent with cold pulses regularly reaching 10 m depth between September and December 2015 (fig. S1B). Coupled with the strong correlation between cold pulse occurrence at 25 and 15 m over this period (Pearson’s *r* = 0.82, *P* < 0.001), our observations imply that cold pulses were influencing corals and macroalgae at 10-m-depth atoll wide (Figs. 1 to 4).

Hydrodynamic processes acting at multiple scales likely contribute to the high spatial and temporal variation of coral bleaching during heatwaves. The complexity of current flows and upwelling around reefs can generate localized variability in nutritional resources (*17*), temperature (*40*), and thermal refugia (*6*), which independently or collectively influence bleaching outcomes. At Palmyra during the period of peak upwelling (September to November 2015), there was notably less cooling at 15 m compared to 25 m depth (Fig. 4A). At 15 m depth, a cooling of <1°C occurred for 93% of the peak upwelling period, with drops of >2°C occurring for only 1.7% of the time (Fig. 4B). In contrast at 25 m depth, cooling of >1°C occurred for 31% of the time, with cooling of >2°C occurring more than nine times more often than at 15 m (Fig. 4B). Collectively, these results show that upwelling likely has a meaningful cooling effect below 15 m depth but that the thermal refuge provided by upwelling rapidly declines toward the surface at Palmyra. Our sampled corals and prior bleaching observations (*39*) were limited to 10 m depth, and, therefore, upwelling-induced cooling during the period of peak thermal stress likely did not play a major role in coral survival in 2015. The current understanding of how high-frequency exposure to rapid, short duration pulses of cooler water influences coral thermal stress remains limited. Thus, while increased food supply is likely the primary benefit associated with upwelling during this event, we cannot rule out potential physiological benefits associated with high-frequency cold pulses that reduced overall water temperature by <1°C. Determining the regional- and local-scale physical drivers that promote coral survival through increased food supply, protective cooling, or a combination of both will help identify coral reef areas that might be more sheltered from future marine heat waves.

### Major El Niño events modify Pacific Ocean currents and enhance localized upwelling

**Fig. 5.**
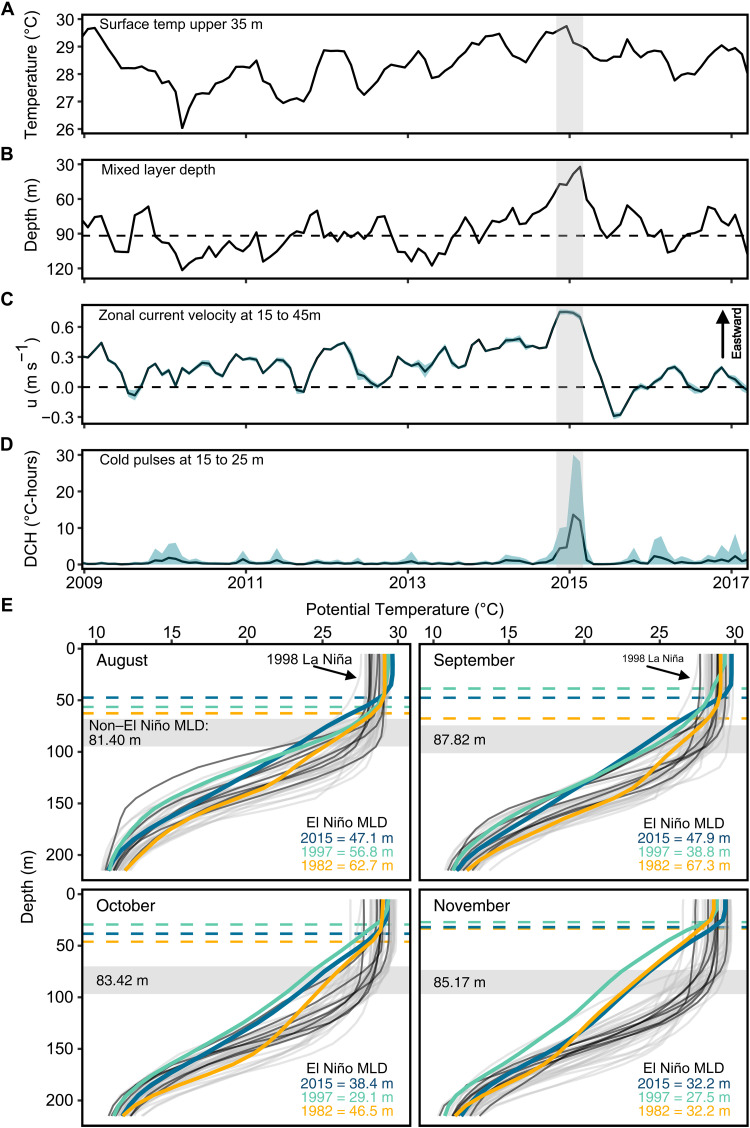
Enhanced upwelling on Palmyra occurs during strong El Niños and is associated with a pronounced shoaling of the surface mixed layer and increased flow of the eastward flowing North Equatorial Counter Current (NECC). (**A**) Monthly mean temperature in the upper 35 m of the water column. (**B**) Monthly mixed layer depth (MLD). Dashed line denotes the long-term mean excluding 2015 (91.9 m ± 13.8 SD). (**C**) Monthly mean zonal current velocity (u) in the upper 45 m of the water column (shading, ±1 SD). (**D**) Monthly mean DCHs (±1 SD) between 15 and 25 m depth. Gray bars indicate August to November 2015. (**E**) Temperature profiles of the upper water column. Colored lines and text represent major El Niños (1982, 1997, and 2015) and their corresponding mean MLDs (dashed lines). Black lines represent non–El Niño years with *Halimeda* δ^15^N data, and gray lines depict all other years from 1980 to 2020. Shaded horizontal regions depict monthly mean MLD ± 1 SD for all years except the major El Niños and the 1998 La Niña (arrow). The MLD on Palmyra during moderate El Niños is stable and below 80 m. By contrast, during each major El Niño, the MLD is substantially shallower (29 to 67 m) and progressively shoals from August to November, which coincides with the peak of upwelling. See fig. S6 and tables S6 and S7 for data between 1980 and 2020 and table S6 and S7 for metadata from NOAA temperature loggers used to quantify cold pulses.

**Fig. 6.**
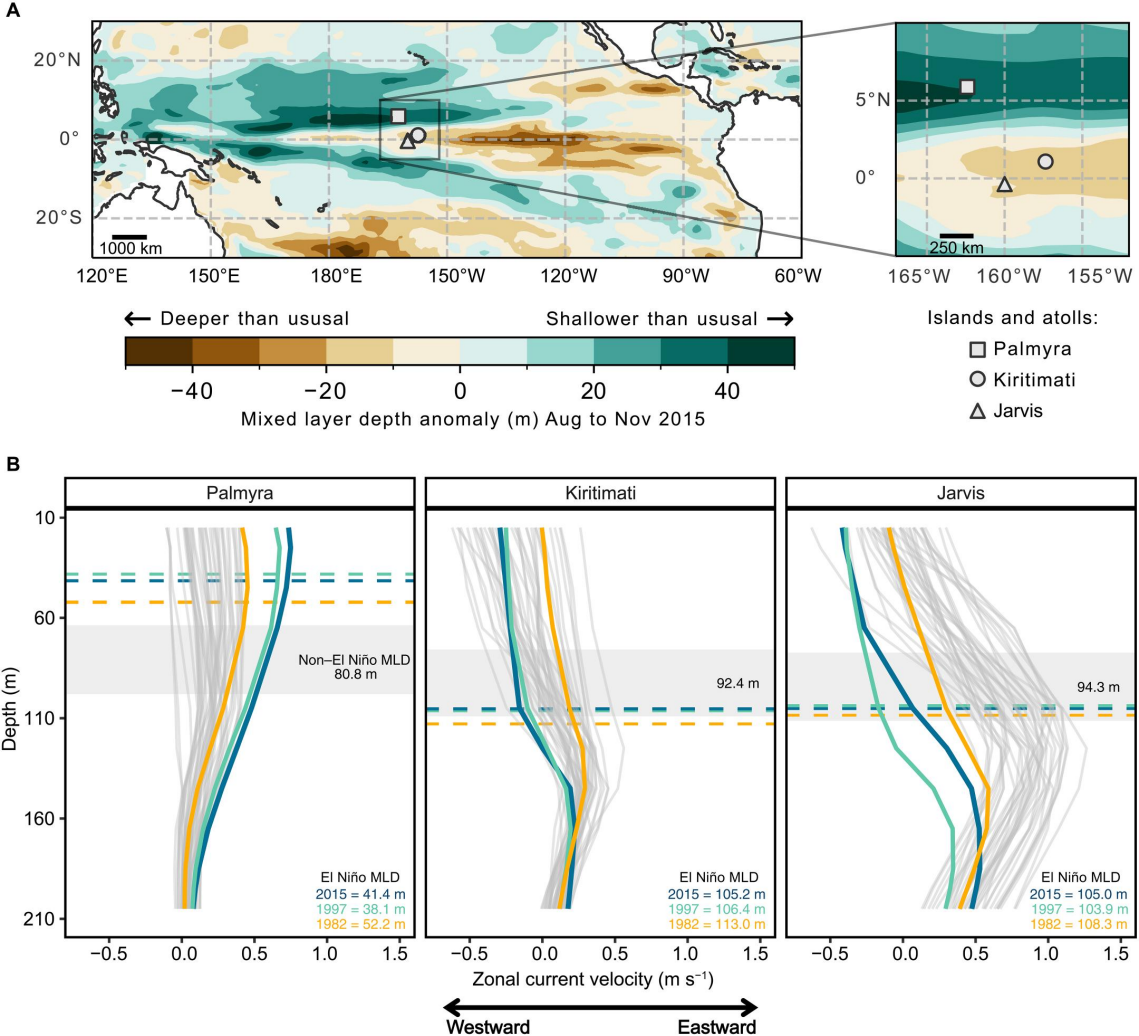
Regional patterns of mixed layer depth (MLD) anomalies and current velocities reveal how oceanographic drivers facilitate contrasting patterns of coral survival. (**A**) MLD anomalies across the Pacific from August to November 2015 relative to the period 1980–2020. The inset highlights the stark contrast in mixed layer shoaling during the 2015 El Niño at Palmyra where coral survival was high compared to nearby Kiritimati and Jarvis where coral mortality was extreme during 2015. (**B**) Zonal current velocity (u) profiles of the upper water column at Palmyra, Kiritimati, and Jarvis. These profiles reveal the important interaction between a shallower MLD and zonal current velocities that drives an increase (or cessation) of upwelling during major El Niños in the central Pacific. At Palmyra, the eastward acceleration of the NECC coincided with a shallower mixed layer leading to increased upwelling. By contrast, the eastward flowing equatorial undercurrent (EUC) that facilitates equatorial upwelling at Kiritimati and Jarvis nearly shuts down during major El Niños. Colored lines and text represent major El Niños (1982, 1997, and 2015) and their corresponding mean MLDs between August and November (dashed lines). Gray lines depict all other years from 1980 to 2020. Shaded horizontal regions depict monthly mean MLD ± 1 SD for all years except the major El Niños.

We used 40 years (1980–2020) of temperature data, surface isothermal depth estimates, and zonal current velocities from the National Centers for Environmental Prediction (NCEP) Global Ocean Data Assimilation product (GODAS; see methods and https://psl.noaa.gov/data/gridded/data.godas.html) to examine temporal changes in regional oceanographic conditions in the central Pacific. Increased upwelling during the 2015–2016 El Niño was associated with a shallower mixed layer depth, estimated as the bottom of the isothermal layer (0.8°C difference from the surface) (*41*), and a strengthening of the NECC. During the 2015–2016 El Niño, the eastward flow of the NECC in the upper 100 m of the water column increased fourfold between August and November relative to the long-term mean during this period (Figs. 5A and 6B). The eastward flow acceleration coupled with a pronounced shoaling of the surface mixed layer (Fig. 5B) reduced the density barrier to upwelling. These regional-scale changes, driven by ocean and atmospheric warming during El Niño, facilitated island-wide upwelling (Figs. 3 and 5, C and D). The interaction between strong NECC flow and Palmyra’s topography can generate localized vertical isotherm displacement and a shallower mixed layer near the island (*42*), facilitating the injection of nutritional resources across the thermocline and into the depths of shallow coral communities. Over the past 40 years, increased NECC strength and a concurrent shoaling of the surface mixed layer only occurred at Palmyra during the major El Niños of 1982–1983, 1997–1998, and 2015–2016, with mixed layers up to 60% shallower relative to non–El Niño years (Fig. 5E). This reveals a previously unknown connection between regional- and local-scale oceanographic processes that bolster nutritional resource supply to Palmyra’s corals during the most severe marine heatwaves.

Localized upwelling driven by island-current interactions also influences coral communities at the nearby islands of Kiritimati and Jarvis (~700 km south of Palmyra) (*7*, *43*, *44*). During the 2015 El Niño, mixed layer depths are altered across the Pacific leading to areas of anomalously shallow mixed layers off the equator (Fig. 6A). Regional current dynamics are also modified during El Niño. Most notably, the EUC effectively shuts down during the strong El Niños (Fig. 6B) (*34*, *45*), leading to a cessation of equatorial upwelling. Coupled with extreme thermal stress, this loss of upwelling contributed to severe coral mortality at Kiritimati and Jarvis in 2015–2016 (*15*, *46*). In contrast, the combination of a shallower mixed layer depth and increased NECC flow at Palmyra enhanced upwelling (Figs. 5 and 6) and facilitated high coral survivorship (*39*). These observations reveal how oceanography and island geography can contribute to stark differences in coral survival at neighboring islands.

A central challenge in coral reef science is disentangling how natural physical drivers and anthropogenic drivers interact across scales and background conditions to influence differences in ecosystem response to bleaching (*47*). While many reefs are showing signs of rapid change and reorganization following repeated mass bleaching, some like Palmyra appear to positively deviate from the global trend (*39*, *48*). Our results underscore the need to consider the connected roles of regional and local oceanographic processes in driving on-reef biological mechanisms that may underpin such coral reef “bright spots.” Under continued ocean warming, changes to large-scale physical processes might benefit reefs in certain areas. Understanding these biophysical connections and how they may change in the future is vital if we are to accurately predict spatial and temporal variations in reef vulnerability to climate change.

## MATERIALS AND METHODS

### Coral stable isotope analyses

To examine temporal changes in coral trophic ecology, we collected top-central branch tips from unshaded *P. meandrina* colonies at four long-term monitoring sites (10 m depth) on Palmyra Atoll, central Pacific (fig. S1A). We sampled in September 2012 (*n* = 10 at all sites), 2014 (*n* = 10 from two sites), and 2015 (*n* = 10 at all sites). In 2015, nine additional sites were sampled (*n* = 5 per site, 10 m depth) (*49*) and used here to highlight the atoll-wide isotopic niche compression in *P. meandrina* relative to non–El Niño years (fig. S3). Samples were transported on ice and frozen at −20°C. In the laboratory, the coral host and endosymbiont were separated via centrifugation (*17*, *49*) and loaded on pre-combusted GF/F filters. Samples were briefly acidified with dropwise addition of 5% HCl to remove CaCO_3_ and dried at 60°C for 48 hours. Bulk δ^13^C, δ^15^N, and mass percent C and N (fig. S1) were measured via EA-IRMS with Costech elemental analyzer (Valencia, CA, USA) coupled to a Thermo Scientific Delta XP Plus isotope ratio mass spectrometer (Bremen, Germany). Isotopic data are reported as delta values (δ) and expressed in ‰ relative to the international standards (Vienna Pee Dee Belemnite) and atmospheric N_2_ for carbon and nitrogen, respectively. Within-run SD of in-house reference material was <0.2‰ for δ^13^C and δ^15^N.

### Macroalgal stable isotope analyses

The pantropical reef alga, *H. opuntia*, can reliably distinguish on-reef nitrogen sources at intra-island scales (*17*). To investigate interannual variation in nitrogen supply to Palmyra, we compiled an 8-year time series of δ^15^N values from *H. opuntia* (Fig. 2B) between 2005 and 2017. We acquired mean ± 95% confidence intervals (CI) δ^15^N values from the literature for 2008 (*50*) and 2010 (*51*). In all years, except 2008, samples were collected from 10 m depth on Palmyra’s fore reefs from two (2014 and 2016: *n* = 5 per site, *n* = 10 total) or all four of the coral collection sites (2005, 2010, 2012, 2015, 2017: *n* = 5 per site, *n* = 20 total; fig. S5A). The low δ^15^N value in 2008 may be an underestimate of fore reef δ^15^N values due to inclusion of samples collected in back reef or reef terrace habitats (*50*), which have lower δ^15^N because of high connectivity to the lagoon (*17*).

In all years, *Halimeda* was transported on ice and frozen at −20°C before the upper three to four segments on three different branch tips were removed, cleaned of epiphytes, decalcified in 5% HCl, and dried for 48 to 72 hours at 60°C. The samples were the homogenized with a ball mill and analyzed for δ^13^C, δ^15^N, and percent mass C and N as above. Decalcification does not bias δ^15^N values of *Halimeda kanaloana* (*52*), and we found no difference in mean δ^15^N values of paired acidified (13.4‰ ± 0.4 SD) versus nonacidified (13.5‰ ± 0.3 SD) samples of *Halimeda* sp. collected at 10 m depth on Starbuck Island, Southern Line Islands (*n* = 5). See Supplementary Materials and Methods for information on lagoon transplants to address the possibility of N_2_ fixation on Palmyra during 2015.

### Inorganic nutrient analysis

Discrete water samples were collected over multiple days at 10 m depth at the four main sampling sites in 2012 (*n* = 13) and the four main sites plus nine additional sites spanning the entire north and south shores of Palmyra in 2015 (*n* = 13 sites, 22 samples). Individual samples were filtered through GF/F filters (0.7 μm) and frozen at −20°C before analysis. Samples were analyzed for nitrate and nitrite in the Analytical Lab at the Marine Science Institute, UC Santa Barbara (2012) and the Analytical Core Lab at King Abdullah University of Science and Technology (KAUST) (2015).

### Remote sensing of surface ocean chlorophyll *a* concentrations

To examine temporal changes in surface ocean production across the Northern Line Islands, we used the 8-day 0.0147° (~4-km) spatial resolution chlorophyll *a* (mg m^−3^) product derived from the Moderate Resolution Imaging Spectroradiometer (https://modis.gsfc.nasa.gov) (*1*, *11*). The typical conditions for the region from May to September (peak productivity to sample collection) were averaged across all sampling years before 2015 and contrasted during the 2015 El Niño and in 2017 when oceanographic conditions returned to climatological norms. To link regional fluctuations in primary productivity with on-reef variation in δ^15^N values, we compared *Halimeda* δ^15^N with monthly mean surface chlorophyll *a* estimates at the time of collection (September), at 1-to-4-month lags before sample collection, and to the average surface chlorophyll *a* concentration over 4 months before sampling.

### Cold pulse detections and quantification

Upwelling events were quantified using a TSI developed on Palmyra (*38*). This method is optimized to detect and quantify upwelling-induced cold pulses in warm, weakly stratified coral reef environments and requires at least two temperature loggers to be used: one at the target depth of cold pulse detection and a shallower one. For example, cold pulses can be estimated at 25 m depth using loggers from 25 and 15 m and at 15 m depth using loggers at 15 and 5 m. The upslope propagation of cold pulses leads to anomalous TSI values, due to the difference in magnitude or the lag in temperature drops between the two loggers. The TSI is then compared to a locally relevant threshold [sensu (*37*)], and cold pulses are detected as continuous periods of TSI above this threshold.

Cold pulses were quantified as instantaneous DHCs, which correspond to the temperature drops induced by the events multiplied by their duration. This metric accounts for both the duration and magnitude of cold pulses affecting a certain depth. Instantaneous DCHs were then summed over each day to estimate the cumulative impact of cold pulses on the reef slope each day, hereafter referred to as DCH.

We used temperature records collected by the National Oceanic and Atmospheric Administration Pacific Islands Fisheries Science Center’s Pacific Reef and Monitoring Program using Sea-Bird Electronics subsurface temperature recorders (SBE 56; sampling accuracy, 0.002°C) (*53*, *54*). Temperature measurements were collected between 5 and 35 m depth on the outer reef slope between 2009 and 2018 (table S6). For cold pulse activity in 2015, we focused on four temperature records from the north, east, south, and west extremes of Palmyra’s outer reef slope (fig. S5A and table S7). We quantified monthly mean DCH at 15 and 25 m depth for the 3 months leading up to our sample collection in late September. We then calculated % DCH enhancement during the 2015 El Niño relative to the baseline monthly mean DCH during non–El Niño years between 2012 and 2017 (Fig. 3).

To extend the cold pulse record to 2009 and compare with interannual variation in mixed layer depth and current velocity, we calculated monthly mean DCH between 15 and 25 m using all available temperature measurements from the outer reef slope of Palmyra (Fig. 4C and table S6). In addition, in situ temperature measurements from our North sampling site revealed high frequency temperature drops between September and December 2015 (10 m depth; fig. S5B), providing empirical evidence of cold pulses influencing the benthic communities at our sampling depth. Temperature measurements were made using (SeaFET Durafet III with 30 min of sampling frequency and accuracy of ±0.01°C) and corrected to measurements from Sea-Bird SBE 37 microcats (*39*).

Last, we considered the potential cooling effect of cold pulses on Palmyra’s outer reef slope by examining the percentage of time in each day that was influenced by cold pulses of varying magnitude at 15 and 25 m depth. For this, we used data from the site on Palmyra’s north shore as it was the site with the greatest upwelling estimates for both depths. We pooled pulses into four categories on the basis of their magnitude of temperature drop (<1°C, 1° to 2°C, 2° to 3°C, or >3°C). While we cannot explicitly calculate how much cooling was provided to the corals at 10 m by upwelling (our sampling depth and observations of bleaching), this analysis revealed a stark reduction in cold pulse influence on seawater temperature between 25 and 15 m. We also quantified the total percentage of pulses of each magnitude for the 3 months with the greatest upwelling (September, October, and November) to contrast the overall mean conditions cold pulses induced at different depths.

### Regional temperature, mixed layer depth, and current velocity

We used the NCEP GODAS (*55*) provided by National Oceanic and Atmospheric Administration Physical Sciences Laboratory (NOAA PSL), Boulder, CO, USA, and available at https://psl.noaa.gov/data/gridded/data.godas.html to assess variation in the mixed layer depth and zonal current velocity near Palmyra. We averaged the monthly means of potential temperature, isothermal depth, and zonal current velocity from the grid cells on the east and west side of Palmyra (available at a resolution of 0.33° latitude × 1° longitude) between 1980 and 2020. Palmyra lies at the center of the two grid cells, which we averaged together to better reflect regional conditions. The isothermal depth estimates the bottom of the surface mixed layer and is best approximated as a temperature difference of 0.8°C from the surface (*41*). Shallower mixed layers are more conducive to upwelling on Palmyra’s reef slope (*42*). We considered the temporal variability in both the monthly mean temperature of the upper 35 m and the temperature profile of the upper 225 m of the water column. Zonal current velocity near Palmyra reflects the NECC flow past the atoll and is strongest in the upper 100 m of the water column during boreal fall (*42*). We examined interannual variability in peak eastward velocities between in the upper 45 m of the water column between August and November to reflect conditions most relevant to our sampling period and the peak thermal stress on Palmyra.

We also examined regional patterns of mixed layer depth and zonal current velocities to provide insight into the contrasting patterns of coral survival during the 2015 bleaching event at Palmyra and the nearby islands of Kiritimati and Jarvis in the Northern Line Islands. Mixed layer depth anomalies in 2015 were calculated for the average depth during August to November between 1980 and 2020. Zonal current velocities for the upper 205 m of the water column were quantified to capture changes in the deeper EUC that affects the more equatorial islands of Kiritimati and Jarvis.

### Statistical analyses

We compared annual changes in the bulk tissue δ^13^C, δ^15^N, and C:N values of coral host, endosymbionts, and their difference (δ^13^C/δ^15^N_host_ − δ^13^C/δ^15^N_sym_) using linear mixed effects models with year as a fixed factor and site as a random effect (*56*). Assumptions of homogeneity of variance and normal distribution were confirmed through visual inspection of the residuals. In addition, we used bootstrap resampling to consider the magnitude of change in the average coral and endosymbiont δ^13^C, δ^15^N, and C:N values between pre–El Niño (2012) and El Niño (2015) conditions. Data for each tissue fraction and year were randomly sampled with replacement to calculate the difference in mean values between 2012 and 2015. This process was repeated 10,000 times to generate a distribution of mean differences between years, from which we estimated the mean change ± 95% CI. We quantified changes in the isotopic niche of *Pocillopora* using Stable Isotope Bayesian Ellipses (*25*). We estimated interannual variation in niche area using Bayesian ellipse area (SEA_b_) and the standardized ellipse area corrected for sample size (SEA_c_). Furthermore, we calculated proportional changes in the six Layman metrics (*24*) to examine how the isotopic niches of host and endosymbionts were compressed from 2012 (*n* = 38 per fraction) to 2015 (*n* = 39 per fraction).

We estimated changes in the relative reliance on heterotrophy versus autotrophy as the proportional overlap between host and symbiont SEA_c_ (*26*). Significant differences in isotopic position of host and symbiont niches within years and across years for each tissue were determined using a residual permutation procedure and Hotelling *T*^2^ test on the Euclidean distance between centroids (*57*). Quantifying coral trophic strategy using SEA_c_ overlap may overestimate contributions of heterotrophy (*26*). To estimate the magnitude of change in coral trophic strategy during 2015 more conservatively, we estimated mean proportional SEA_c_ overlap values and the associated error to using a bootstrap resampling procedure. See Supplementary Materials and Methods for details.

We modeled interannual variation in *Halimeda* bulk tissue δ^13^C, δ^15^N, and C:N from 2012 to 2017 using linear mixed effects models with year as a fixed factor and site as a random effect. We modeled the relationship between *Halimeda* δ^15^N and nearshore chlorophyll *a* using standard major axis linear regression to identify the lag time between off-reef primary production and local δ^15^N values. Temporal autocorrelation in *Halimeda* δ^15^N values was tested using a generalized least squares model with a first-order autoregressive term (AR1) to control for correlated error structure, but this did not improve on the null model [gls versus gls + AR1, analysis of variance (ANOVA) = 0.8, ΔAIC < 2]. All statistical analyses were performed in R version 4.0.2 (*58*).
