# Supplementary material for: Ocean currents magnify upwelling and deliver nutritional subsidies to reef-building corals during El Niño heatwaves
Source: Sci Adv. 2023 Jun 14;9(24):eadd5032. doi: 10.1126/sciadv.add5032 (PMC10266739; doi:10.1126/sciadv.add5032)
Supplement: Supplementary file 1 — Materials and Methods Figs. S1 to S6 Tables S1 to S7 [file sciadv.add5032_sm.pdf]

Science Advances
AAAS

Supplementary Materials for

Ocean currents magnify upwelling and deliver nutritional subsidies to reef-building corals during El Niño heatwaves

Michael D. Fox *et al.*

Corresponding author: Michael D. Fox, michael.fox@kaust.edu.sa

Sci. Adv. **9**, eadd5032 (2023)
DOI: 10.1126/sciadv.add5032

This PDF file includes:

Materials and Methods
Figs. S1 to S6
Tables S1 to S7

Materials and Methods

Supplemental Methods

Coral isotopic niche overlap resampling

We modified the niche overlap approach from (25) to quantify coral trophic strategies more conservatively. This new approach accounts for shifts in the $\delta^{13}C$ and $\delta^{15}N$ baselines between years and the stark reduction in spatial and individual-level variation during 2015, which constrained the possible Euclidean distance between host and symbiont centroids. In each year, we randomly sampled host and endosymbiont data (with replacement) to maximize the variation in possible SEA_c and position. For each randomly generated set of host and endosymbiont SEA_c we estimated the % SEAc overlap. This was repeated 10,000 times to generate a distribution of possible % SEA_c overlaps, from which we estimated the mean overlap and 75% and 95%CIs for each time period. Sample size in 2014 (n=10) is too low for robust SEA_c estimates, however, these data are shown for transparency. We also present SEA_C overlap estimates from a pooled dataset that includes samples from both 2012 and 2014. This most accurately represents the true niche diversity around Palmyra and provides a conservative baseline trophic strategy for the community for comparison to 2015. By estimating the range of possible SEA_c % overlap values, we present a more realistic estimate of the true trophic continuum of a coral population.

Interestingly, measurements made from this same population using $\delta^{13}C$ analysis of essential amino acids ($\delta^{13}C_{AAess}$) also observed no spatial structure in coral trophic strategy and estimated a high contribution of heterotrophic nutrition to the community as a whole, with 21-53% of colonies relying more on heterotrophy than autotrophy (49). Nevertheless, these methods revealed contrasting pictures of individual-level trophic diversity, with the $\delta^{13}C_{AAess}$ data highlighting intra-colony trophic diversity that is not apparent in the isotopic niches shown here.

Thus, estimates of coral trophic strategy based on isotopic niches may reflect average community-level changes in trophic ecology but cannot capture differences among individuals or at small spatial scales.

Halimeda transplants

During non-El Niño conditions, the $\delta^{15}N$ value of primary producers in Palmyra's lagoon is lower than the outer reef slope by ~2-3 ‰, which was affirmed using a 2-week transplantation of *Halimeda* in 2014 (17). The stark reduction of baseline $\delta^{15}N$ during 2015 may therefore have been associated with a local increase of nitrogen fixation ($\delta^{15}N$ ~ 0 ‰) in Palmyra's lagoon or shallow reef flats. In 2015, we tested for a reduction of *Halimeda* $\delta^{15}N$ values in lagoon and backreef habitats relative to samples collected from the outer reef slope using transplants. Individuals were collected from the back reef near the SW sampling site (Fig. S1A) and deployed across twelve sites spanning Palmyra's west, central, and eastern lagoons (n=2-3 per site) for 3 weeks in September 2015 following (17). This provided an additional week of time compared to 14-day experiment in 2014 that produced a significant change in *Halimeda* $\delta^{15}N$ transplanted in the lagoon (17). Therefore, if Palmyra's lagoon or shallow reef nitrogen pools were dominated by N_2 fixation in 2015, a significant decline in transplanted *Halimeda* $\delta^{15}N$ values would be expected. After three-weeks, we pooled samples across all transplant sites (n= 35) to obtain an average $\delta^{15}N$ value for Palmyra's lagoon and compared to the $\delta^{15}N$ value of the same individuals collected in the backreef. We found no significant change of Halimeda $\delta^{15}N$ values in either habitat. Instead, the mean value of *Halimeda* pre ($\delta^{15}N$ = 6.5 ‰ ± 0.6SD) and post-transplant ($\delta^{15}N$ = 6.7 ‰ ±0.3) did not differ and both were higher than the outer reef slope, contrary to pattens during non-El Niño conditions (17).

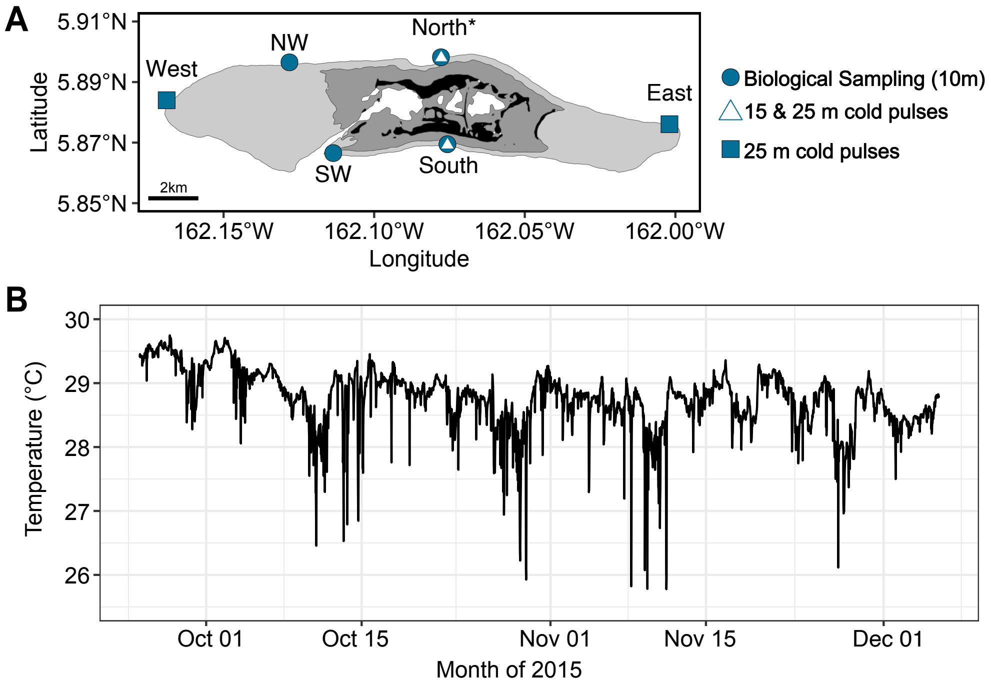

Figure S1 | Long-term ecological and oceanographic monitoring at Palmyra Atoll reveal biophysical connections between coral trophic ecology and El Niño upwelling. A) Palmyra Atoll showing locations of biological collections (circles) and the locations of cold pulse detections at different depths (triangles and squares**).** Land is shown in black with the reef flat and upper fore reef slope outlined in dark and light gray, respectively. At the north and south locations, biological sampling was paired with the STR locations. *In situ* temperature measurements (10 m depth) were recorded at the North biological sampling site (*). B) *In situ* temperature measurements between 24 September and 05 December 2015 at the North site. Temperature was measured using a SeaFET outfitted with a Durafet III combination electrode (30 min sampling frequency) and corrected to measurements from an SBE37 microcat (±0.01 °C). See Fox et al. (39) for details. The high frequency temperature drops are consistent with cold pulses and confirm their influence on reef organisms at our 10 m sampling depth.

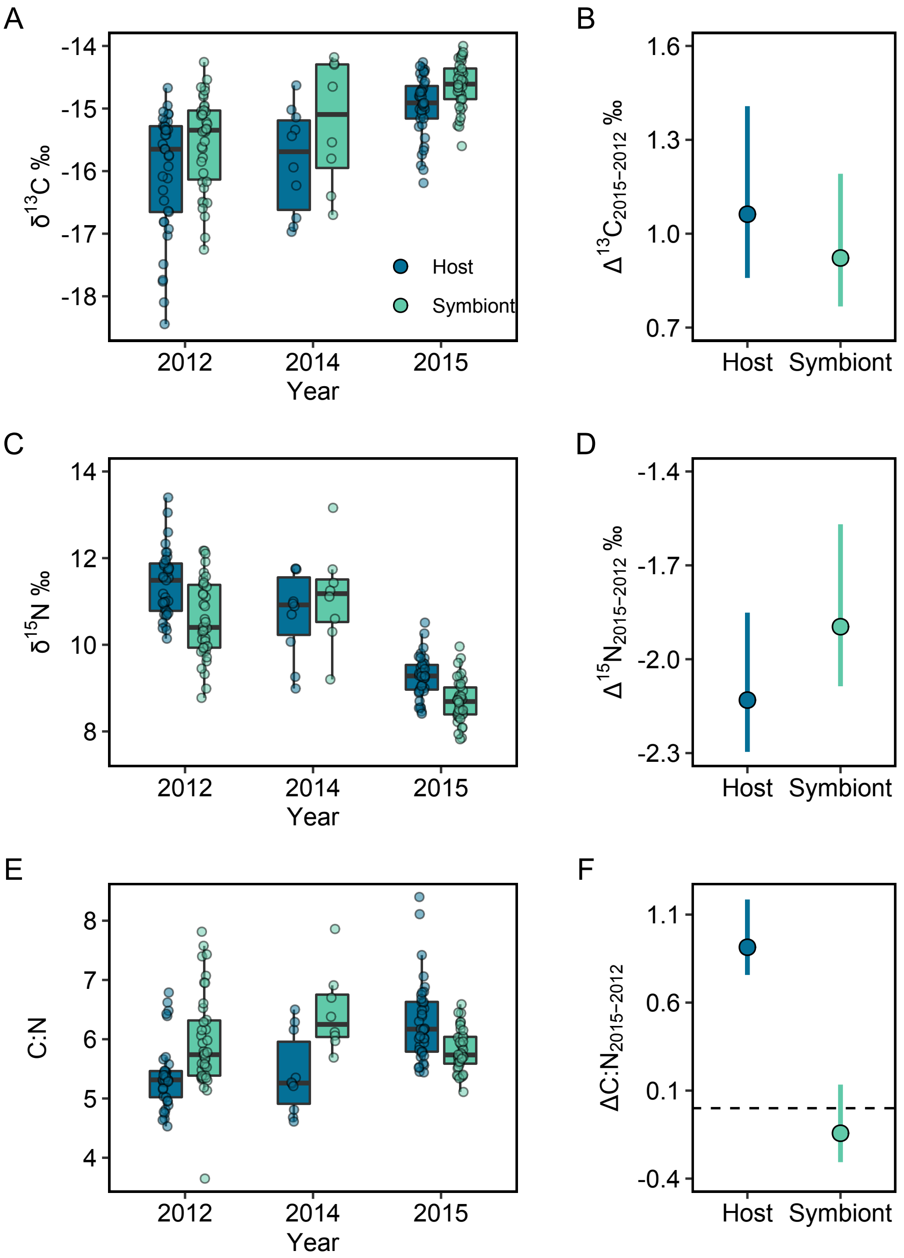

Figure S2 | Changes in coral and endosymbiont bulk tissue $\delta^{13}C$, $\delta^{15}N$, and C:N values of *Pocillopora meandrina.* **A,C,E**) Boxplots illustrate the distribution of all individual colonies (points) sampled across the four monitoring sites (n=2 sites in 2014). Boxes represent the 25th to 75th quartiles and whiskers reflect 1.5x the interquartile range. Raw data are shown as points. Significant differences across years for each tissue fraction are shown with letters ($p<0.05$) and * denotes differences between fractions within years. **B,D,F)** Mean change ±95% CI for each metric between 2015 and 2012 estimated via bootstrap resampling (n=10,000). Values not overlapping 0 reflect significant changes through at $\alpha = 0.05$.

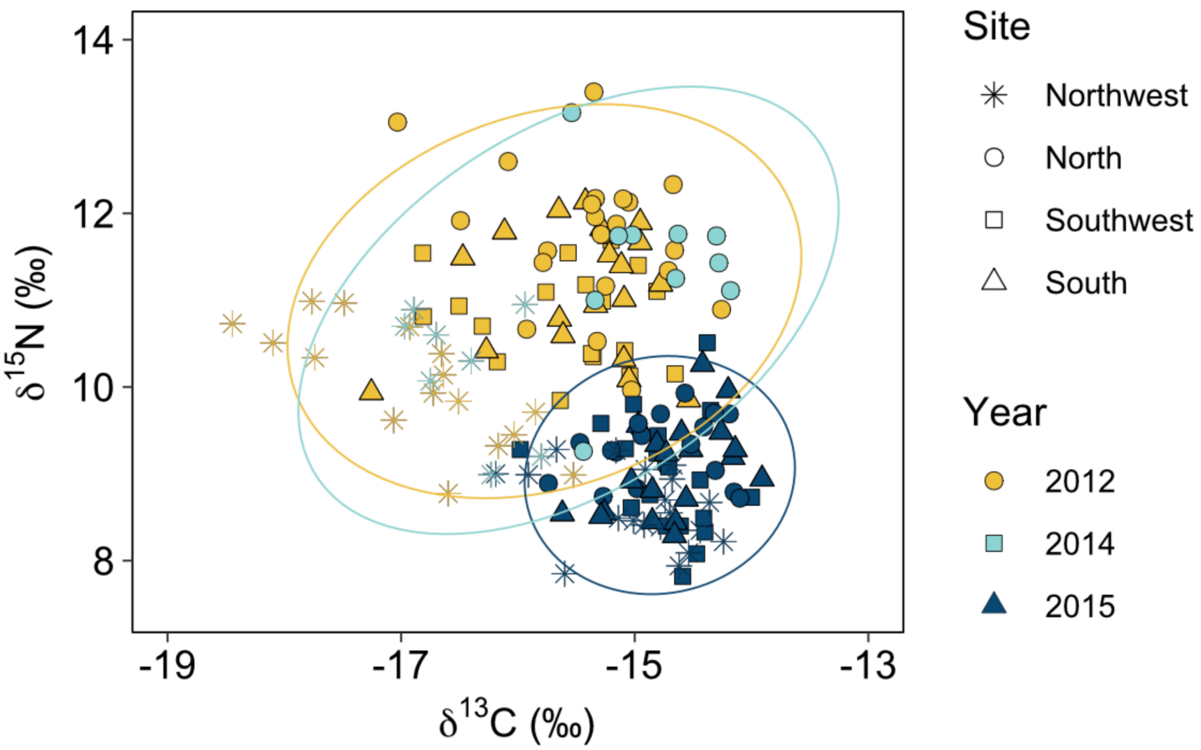

Figure S3 | Spatial homogenization of coral isotopic niches around Palmyra during the 2015 El Niño. Sites around Palmyra differ in resource supply, contributing to site-level variation that is consistent in 2012 and 2014 but not in 2015. Shapes denote coral and endosymbiont values (not differentiated) from long-term monitoring locations (see Fig S5). Ellipses reflect 95% CI (not SEAc) to emphasize magnitude of niche compression in 2015.

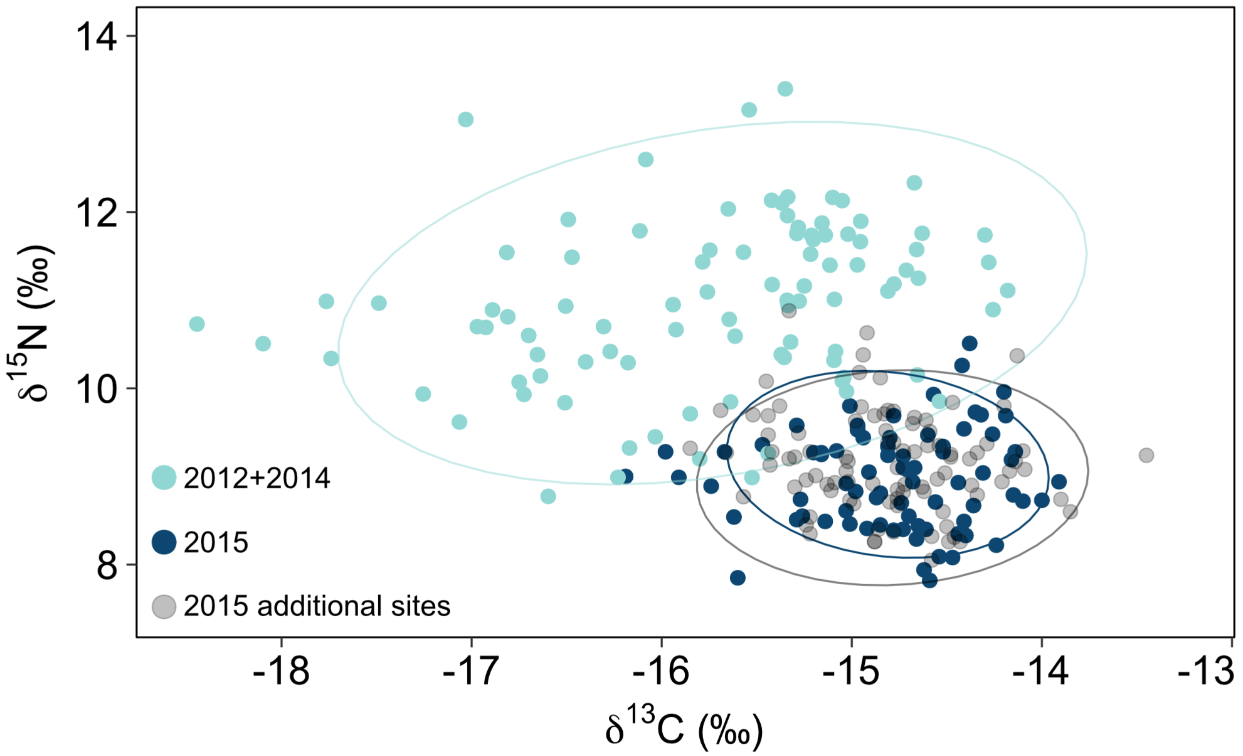

Figure S4 | Spatial homogenization of coral isotopic niches was universal around Palmyra in 2015. Pooled coral and endosymbiont data for 2012 and 2014 from the long-term monitoring sites (light blue) highlight the baseline size of *Pocillopora's* isotopic niche relative to 2015 (dark blue). In 2015, 9 additional sites (10m depth fore reef) were sampled to encompass data from all around the atoll (gray). The inclusion of these additional sites (n=47 per tissue) emphasizes the stark compression of the isotopic niche around the atoll. Ellipses represent 95% CI (not SEAc) for each group.

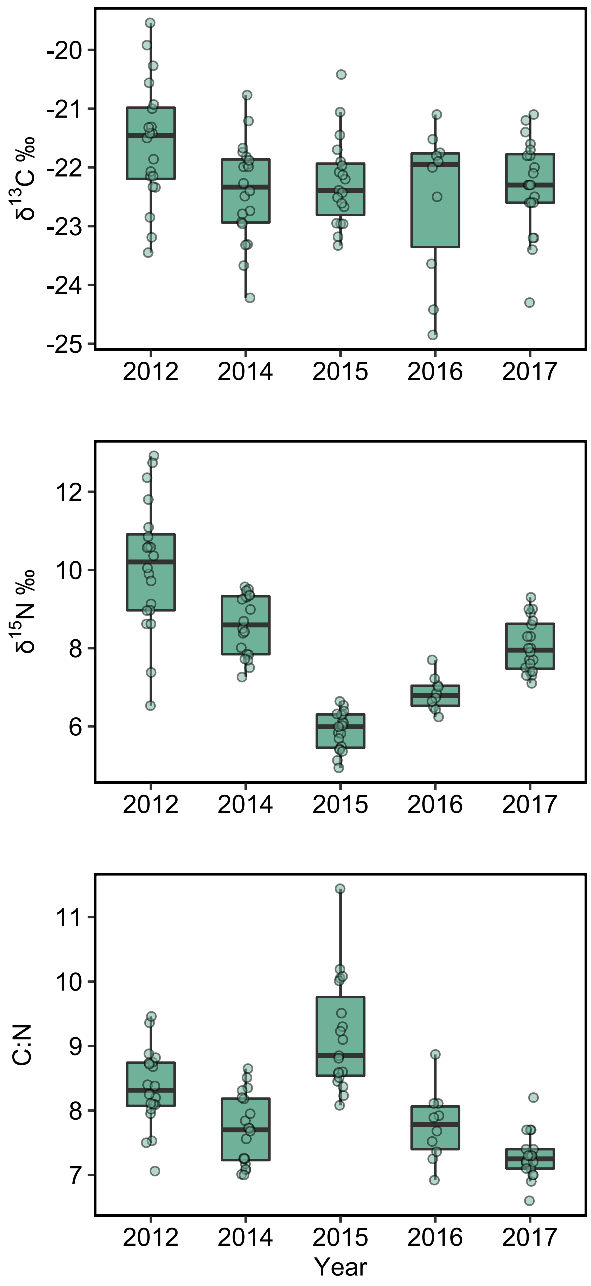

Figure S5 | Interannual variation in *Halimeda* bulk tissue $\delta^{13}C$, $\delta^{15}N$, and C:N values over five years. Samples were collected from all four long-term monitoring sites (n=5 each, n=20 total) except in 2016 where samples were collected from the NW and SW sites (n=5 each, n=10 total). Boxplots illustrate the distribution of all samples (points). Boxes represent the 25th to 75th quartiles and whiskers reflect 1.5x the interquartile range. Letters denote differences between years at $p<0.05$.

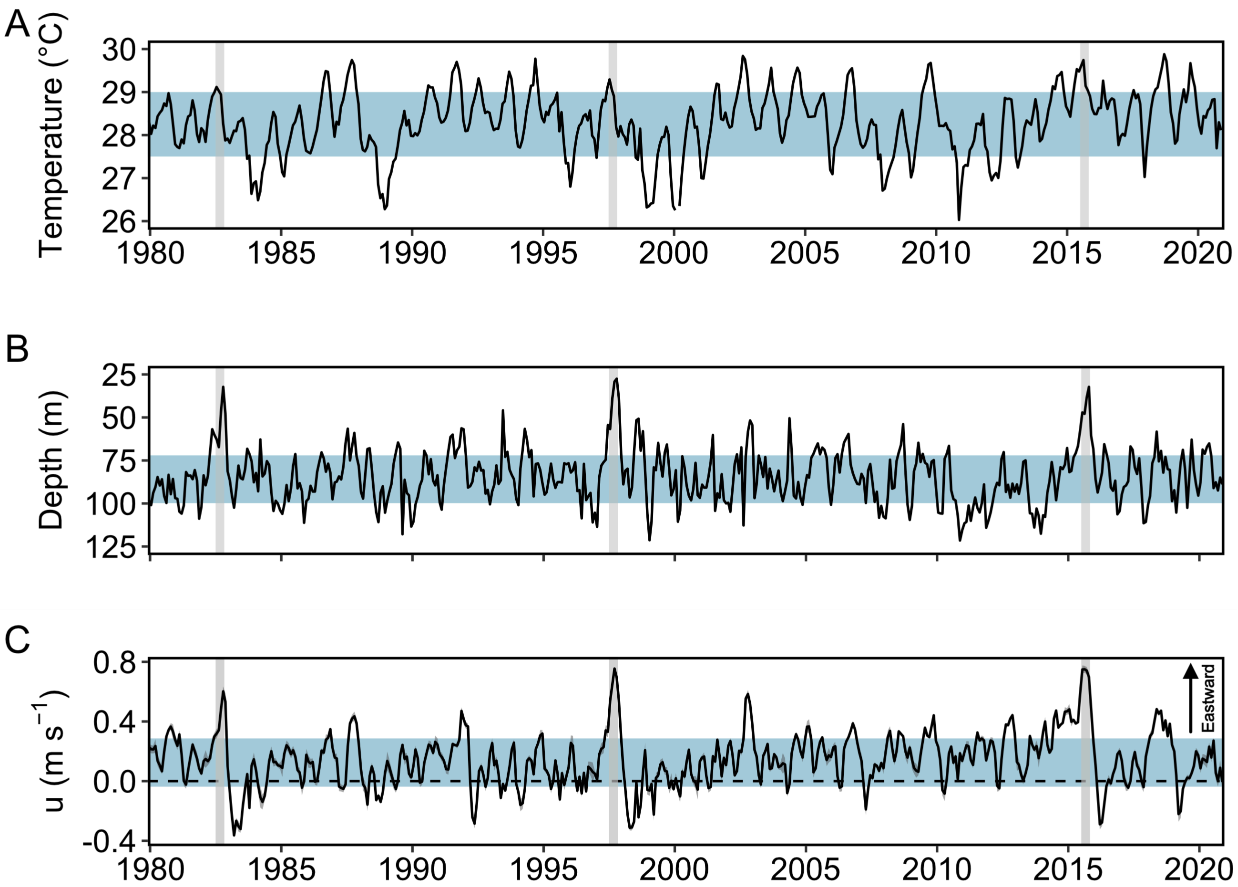

Figure S6 | Oceanographic conditions at Palmyra Atoll over the past 40 years (1980-2020) reveal recurrence of shallow surface mixed layers and accelerated eastward flow during the last three major El Niños. **A**) Surface Temperature in the upper 35 m of the water column, (**B**) depth of the surface mixed layer, and (**C**) mean velocity of the NECC near Palmyra in the upper 45 m of the water column. Shaded blue regions represent long-term mean ± 1SD excluding the major El Niño years. Vertical gray bars denote the last 3 major El Niños. All data are from the National Center for Environmental Prediction Global Ocean Data Assimilation System reanalysis product (NCEP/GODAS; https://psl.noaa.gov/data/gridded/data.godas.html).

Table S1. Statistical summary for the fixed effects of linear mixed effects models comparing changes in coral tissue chemistry between years and tissue fractions. Δ values reflect the difference between coral host and endosymbiont tissues. Significant effects of $p < 0.05$ are in bold. Pairwise contrast are illustrated in Fig. S2. For $\delta^{15}N$, 2012 was greater than 2014.

Variable	Factor	SS	df	F	P
$\delta^{13}C$	**Tissue**	5.1	1	14.1	**<0.001**
	**Year**	39.97	2	55.23	**<0.001**
	Tissue*Year	0.27	2	0.37	0.69
	error	58.3	161		
**$\delta^{15}N$**	**Tissue**	4.24	1	12.86	**<0.001**
	**Year**	159.09	2	241.48	**<0.001**
	**Tissue*Year**	4.05	2	6.14	**<0.001**
	error	53.06	161		
**C:N**	**Tissue**	4.03	1	10.14	**0.002**
	**Year**	5.84	2	7.35	**<0.001**
	**Tissue*Year**	14.54	2	18.29	**<0.001**
	error	64.88	161		
$\Delta^{13}C$	Year	0.89	2	2.28	0.11
	error	15.03	77		
**$\Delta^{15}N$**	**Year**	5.61	2	4.84	**0.01**
	error	45.12	77		

Table S2. Changes in the six Layman metrics reflect strong niche compression in 2015 relative to 2012. The values are shown for each metric in units of ‰. The proportional change between years is shown as % change with negative values reflecting a reduction from 2012 to 2015. See (25) for descriptions of each metric.

Tissue Fraction	Layman Metric	2012	2015	% Change
Coral Host	Distance to Centroid	1.10	0.58	-47.55
	$\delta^{13}C$ range	3.77	1.93	-48.86
	$\delta^{15}N$ range	3.26	2.10	-35.54
	Near Neighbor Distance	0.30	0.17	-45.52
	Sd Nearest Neighbor Distance	0.24	0.09	-62.03
	Total Area	8.40	2.22	-73.53
Endosymbionts	Distance to Centroid	1.06	0.56	-46.94
	$\delta^{13}C$ range	3.00	1.69	-43.63
	$\delta^{15}N$ range	3.40	2.14	-37.00
	Near Neighbor Distance	0.26	0.16	-40.28
	Sd Nearest Neighbor Distance	0.20	0.13	-34.48
	Total Area	6.99	2.31	-67.03

Table S3. Results of a residual permutation procedure and Hotellings T^2 tests to determine significant differences in the relative position of coral and symbiont isotopic niches. Contrasts are shown to compare the position of host and symbiont niches within years and to compare the position of the niches between 2012 and 2015 for each tissue fraction. Larger T^2 values indicate greater separation. Significant differences at the p <0.05 level are shown in bold.

**Contrast**	**Year**	**Hollings T2**	**F**	**P**
Within year comparisons	**2012**	**37.60**	**18.05**	**<0.001**
Host vs. Symbiont	2014	18.05	0.81	0.4
	**2015**	**56.10**	**26.96**	**<0.001**
Comparison between years				
**2012 vs. 2015 Host**	**-**	**392.83**	**188.66**	**<0.001**
**2012 vs. 2015 Symbiont**	**-**	**264.18**	**126.88**	**<0.001**

Table S4. Statistical summary for the fixed effects of linear mixed effects models comparing changes in *Halimeda* tissue chemistry between the years with available raw data (2012-2017). Significant effects of $p < 0.05$ are in bold. Pairwise contrast are indicated by letters in Fig. S5.

Variable	Factor	SS	df	F	P
**$\delta^{13}C$**	**Year**	10.00	4	2.95	**0.03**
	error	71.17	84		
**$\delta^{15}N$**	**Year**	119.63	4	119.65	**<0.001**
	error	33.82	81		
**C:N**	**Year**	40.04	4	31.15	**<0.001**
	error	27.63	86		

Table S5. Summary of simple linear regressions comparing surface ocean chlorophyll-a concentrations near Palmyra to *Halimeda* $\delta^{15}N$ values at different temporal lags. Samples were collected in September (2010 collected in early October) and chlorophyll-a concentrations were lagged backward from the time of collection. Significant regressions are shown in bold.

Time Period	Lag (months)	r2	p
May	−4	0.40	0.09
**June**	**−3**	**0.86**	**<0.01**
July	−2	0.49	0.05
August	−1	0.14	0.35
September	0	0.48	0.06
Mean May-Sept	-	**0.72**	**0.01**

Table S6. Metadata from NOAA temperature logger deployments at Palmyra between 2009 and 2018. The loggers from the four focal locations in the study can be identified using the Logger IDs reported in Table S7. This table reflects all available logger data from Palmyra's outer reef slope that was used to calculate monthly average DCH between 15 and 25 m from 2009 to 2017.

OCC Site ID	Latitude	Longitue	Depth (m)	Region	Island	Instrument Type	Instrument Serial #	Deployment Cruise	Retrevial Cruise	Valid Data Start Date (UTC)	Logger ID	Valid Data End Date (UTC)	Sampling Frequency (Sec)
OCC-PAL-056	5.86089	-162.12671	20.4	PRIA	Palmyra	SBE37	37SM270051940	HI1001_LEGIII	HA1201_LEGIV	14-04-2010 14:40:01	1415	16-05-2011 23:45:03	150
OCC-PAL-007	5.863563	-162.03067	24.6	PRIA	Palmyra	STR	5604690	HA1501_Line	HA1801_Line	16-04-2015 09:34:59	2231	30-03-2017 02:04:59	300
OCC-PAL-046	5.863655	-162.03059	18.9	PRIA	Palmyra	SBE37	37SM321022979	HI1001_LEGIII	HA1201_LEGIV	16-04-2010 04:37:31	1420	16-05-2011 23:45:02	150
OCC-PAL-008	5.86378	-162.03063	15.6	PRIA	Palmyra	STR	39368591648	HI1001_LEGIII	HA1201_LEGIV	04-09-2010 00:57:30	1424	08-08-2011 04:20:00	150
OCC-PAL-008	5.863805	-162.03062	14	PRIA	Palmyra	STR	5604650	HA1501_Line	HA1801_Line	16-04-2015 05:24:59	2230	08-04-2018 03:51:26	300
OCC-PAL-015	5.863967	-162.12696	15.2	PRIA	Palmyra	STR	39331791196	OES0404	HI0604	04-02-2004 00:01:02	211	28-03-2006 00:31:02	1800
OCC-PAL-015	5.864062	-162.12697	15.33	PRIA	Palmyra	STR	39541654739		HI1001_LEGIII	10-06-2009 00:00:00	1241	04-07-2010 19:00:00	1800
OCC-PAL-015	5.864103	-162.12694	15.2	PRIA	Palmyra	STR	5604759	HA1501_Line	HA1801_Line	18-04-2015 03:14:59	2239	08-03-2018 05:29:59	300
OCC-PAL-015	5.864145	-162.12699	15.4	PRIA	Palmyra	STR	5.69066E+11	HA1201_LEGIV	HA1501_Line	15-05-2012 00:01:59	1850	18-04-2015 01:06:59	60
OCC-PAL-015	5.86415	-162.12699	15.5	PRIA	Palmyra	STR	39558874320	HI1001_LEGIII	HA1201_LEGIV	04-07-2010 19:00:00	1410	14-05-2012 21:00:00	1800
OCC-PAL-045	5.864227	-162.03065	9.75	PRIA	Palmyra	STR	39390381819	HI0604	HI0803	27-03-2006 23:20:05	449	03-02-2008 22:50:05	1800
OCC-PAL-008	5.864228	-162.03067	10.68	PRIA	Palmyra	STR	39486894033	HI0803	HI1001_LEGIII	04-02-2008 01:30:00	655	04-09-2010 00:00:00	1800
OCC-PAL-045	5.864258	-162.0307	10.1	PRIA	Palmyra	STR	39432363062	HI1001_LEGIII	HA1201_LEGIV	04-09-2010 00:17:30	1427	22-09-2011 03:37:30	150
OCC-PAL-045	5.864258	-162.0307	10.1	PRIA	Palmyra	STR	39558874426	HI1001_LEGIII	HA1201_LEGIV	04-09-2010 00:30:00	1426	15-05-2012 02:30:00	1800
OCC-PAL-044	5.864452	-162.03177	10.1	PRIA	Palmyra	STR	39368591641	HI1001_LEGIII	HA1201_LEGIV	04-09-2010 08:10:00	1429	11-01-2011 10:07:30	150
OCC-PAL-047	5.866383	-162.11364	19.3	PRIA	Palmyra	STR	39541654695		HI1001_LEGIII	18-09-2009 22:39:00	1238	04-08-2010 20:27:00	90
OCC-PAL-047	5.866403	-162.11366	19.6	PRIA	Palmyra	STR	39541654752	HI1001_LEGIII	HA1201_LEGIV	04-08-2010 20:30:00	1428	15-05-2012 00:00:00	1800
OCC-PAL-049	5.866467	-162.11351	13.61	PRIA	Palmyra	STR	39541654693		HI1001_LEGIII	18-09-2009 22:33:00	1237	04-08-2010 02:19:30	90
OCC-PAL-049	5.866482	-162.11362	14.67	PRIA	Palmyra	STR	39368591563	HI1001_LEGIII		04-08-2010 02:20:00	1407	25-10-2010 23:57:30	150
OCC-PAL-048	5.866544	-162.11277	12.2	PRIA	Palmyra	STR	39390381865	HI1001_LEGIII	HA1201_LEGIV	04-08-2010 20:00:00	1425	15-05-2012 00:00:00	1800
OCC-PAL-048	5.866562	-162.11275	12.19	PRIA	Palmyra	STR	39541654692		HI1001_LEGIII	18-09-2009 22:48:00	1239	04-08-2010 19:33:00	90
OCC-PAL-055	5.866668	-162.12652	10.3	PRIA	Palmyra	STR	39368591564	HI1001_LEGIII	HA1201_LEGIV	04-07-2010 21:22:30	1412	05-03-2011 02:55:00	150
OCC-PAL-027	5.869354	-162.07525	30.2	PRIA	Palmyra	STR	39368591642	HA1201_LEGIV		19-05-2012 12:00:00	1873	28-09-2012 23:38:00	60
OCC-PAL-011	5.869418	-162.07522	25	PRIA	Palmyra	STR	5.69066E+11	HA1201_LEGIV		15-05-2012 22:15:59	1855	28-09-2012 22:36:59	60
OCC-PAL-011	5.869433	-162.07522	24.53	PRIA	Palmyra	STR	39240220358		HA1501_Line	29-09-2012 00:36:51	1893	17-04-2015 19:36:53	3600
OCC-PAL-011	5.869458	-162.07525	24.4	PRIA	Palmyra	STR	5604628	HA1501_Line	HA1801_Line	17-04-2015 23:39:59	2236	07-07-2018 20:44:59	300
OCC-PAL-011	5.869458	-162.07525	24.4	PRIA	Palmyra	STR	5604661		HA1801_Line	19-05-2016 12:04:59	2298	08-06-2018 12:09:59	300
OCC-PAL-032	5.869528	-162.07517	19.5	PRIA	Palmyra	STR	5.69066E+11	HA1201_LEGIV		15-05-2012 21:43:59	1854	28-09-2012 22:51:59	60
OCC-PAL-012	5.869594	-162.07514	14.6	PRIA	Palmyra	STR	5.69066E+11	HA1201_LEGIV		15-05-2012 21:41:59	1853	28-09-2012 23:37:59	60
OCC-PAL-012	5.869598	-162.07513	14.7	PRIA	Palmyra	STR	39390383010		HA1501_Line	28-09-2012 23:50:31	1894	23-02-2015 08:50:31	3600
OCC-PAL-012	5.869606	-162.07511	14.2	PRIA	Palmyra	STR	5604626	HA1501_Line		18-04-2015 02:24:59	2237	18-05-2016 09:49:59	300
OCC-PAL-031	5.869727	-162.07515	10.7	PRIA	Palmyra	STR	5.69066E+11	HA1201_LEGIV		15-05-2012 21:30:59	1852	28-09-2012 23:37:59	60
OCC-PAL-012	5.869736	-162.0755	14.2	PRIA	Palmyra	STR	5604660		HA1801_Line	19-05-2016 15:59:59	2299	08-06-2018 06:54:59	300
OCC-PAL-013	5.870108	-162.07509	5	PRIA	Palmyra	STR	5.69066E+11	HA1201_LEGIV		15-05-2012 21:08:59	1851	28-09-2012 23:37:59	60
OCC-PAL-013	5.870118	-162.0751	5.5	PRIA	Palmyra	STR	39368591669		HA1501_Line	10-02-2012 20:47:21	1895	20-08-2014 09:47:21	3600

Table S6 cont.

OCC-PAL-013	5.870139	-162.07512	4.9	PRIA	Palmyra	STR	5604649	HA1501_Line		18-04-2015 03:14:59	2238	17-05-2016 11:39:59	300
OCC-PAL-006	5.875603	-162.00379	15.2	PRIA	Palmyra	STR	5604623	HA1501_Line	HA1801_Line	16-04-2015 03:14:59	2228	23-11-2017 17:04:59	300
OCC-PAL-006	5.875648	-162.00391	14.8	PRIA	Palmyra	STR	5.69066E+11	HA1201_LEGIV	HA1501_Line	20-05-2012 02:08:59	1877	15-04-2015 19:29:59	60
OCC-PAL-005	5.876062	-162.00196	25.7	PRIA	Palmyra	STR	5604624	HA1501_Line	HA1801_Line	15-04-2015 22:09:59	2227	06-08-2018 23:04:59	300
OCC-PAL-005	5.876094	-162.00198	26	PRIA	Palmyra	STR	5.69066E+11	HA1201_LEGIV	HA1501_Line	19-05-2012 23:21:59	1878	15-04-2015 19:50:59	60
OCC-PAL-034	5.87611	-162.00237	20.6	PRIA	Palmyra	SBE37	37SM275312141	HI1001_LEGIII	HA1201_LEGIV	14-04-2010 14:40:01	1455	16-05-2011 23:45:02	150
OCC-PAL-034	5.87611	-162.00237	21.1	PRIA	Palmyra	STR	39368591674	HI1001_LEGIII	HA1201_LEGIV	13-04-2010 23:00:00	1454	15-05-2012 00:30:00	1800
OCC-PAL-034	5.876111	-162.00237	18.89	PRIA	Palmyra	STR	39486894036	HI0803	HI1001_LEGIII	04-02-2008 22:30:00	661	13-04-2010 22:30:00	1800
OCC-PAL-035	5.876367	-162.00206	32.61	PRIA	Palmyra	STR	39486894043	HI0803	HI1001_LEGIII	04-02-2008 22:00:00	660	04-12-2010 21:00:00	1800
OCC-PAL-035	5.87637	-162.00206	32.5	PRIA	Palmyra	STR	39390381811	HI1001_LEGIII	HA1201_LEGIV	04-12-2010 21:30:00	1453	27-03-2012 21:30:00	1800
OCC-PAL-041	5.882429	-162.033	10.4	PRIA	Palmyra	STR	39368591675	HI1001_LEGIII	HA1201_LEGIV	04-09-2010 19:30:00	1432	16-08-2011 06:32:30	150
OCC-PAL-042	5.882775	-162.03298	14.6	PRIA	Palmyra	STR	39390383025	HI1001_LEGIII	HA1201_LEGIV	04-10-2010 12:00:00	1431	14-09-2011 11:17:30	150
OCC-PAL-043	5.882934	-162.03293	19.6	PRIA	Palmyra	SBE37	37SM296962482	HI1001_LEGIII	HA1201_LEGIV	16-04-2010 04:37:30	1436	16-05-2011 23:45:04	150
OCC-PAL-017	5.883225	-162.13321	14.5	PRIA	Palmyra	STR	5604651	HA1501_Line	HA1801_Line	19-04-2015 00:44:59	2240	28-10-2017 06:54:59	300
OCC-PAL-036	5.883783	-162.16859	19.41	PRIA	Palmyra	STR	39474933545	HI0803	HI1001_LEGIII	31-03-2008 22:30:00	649	04-11-2010 22:00:00	1800
OCC-PAL-036	5.88383	-162.16857	19.2	PRIA	Palmyra	SBE37	37SM305932690	HI1001_LEGIII	HA1201_LEGIV	16-04-2010 04:37:31	1450	16-05-2011 23:45:03	150
OCC-PAL-036	5.88383	-162.16857	19.2	PRIA	Palmyra	STR	39558873987	HI1001_LEGIII	HA1201_LEGIV	04-11-2010 22:30:00	1449	18-05-2012 20:30:00	1800
OCC-PAL-016	5.88398	-162.16889	24.7	PRIA	Palmyra	STR	5604698	HA1501_Line	HA1801_Line	20-04-2015 18:34:59	2242	08-01-2018 23:54:59	300
OCC-PAL-016	5.883987	-162.16889	24.9	PRIA	Palmyra	STR	5.69066E+11	HA1201_LEGIV	HA1501_Line	19-05-2012 03:26:59	1871	19-04-2015 20:13:59	60
OCC-PAL-037	5.884095	-162.16906	32.8	PRIA	Palmyra	STR	39541654761	HI1001_LEGIII	HA1201_LEGIV	04-11-2010 21:30:00	1451	18-05-2012 20:00:00	1800
OCC-PAL-037	5.884125	-162.1691	32.91	PRIA	Palmyra	STR	39486894038	HI0803	HI1001_LEGIII	31-03-2008 22:00:00	648	04-11-2010 21:00:00	1800
OCC-PAL-050	5.896363	-162.12817	11.08	PRIA	Palmyra	STR	39541654756		HI1001_LEGIII	16-09-2009 21:19:30	1234	04-08-2010 00:31:30	90
OCC-PAL-050	5.896388	-162.12813	11.3	PRIA	Palmyra	STR	39368591655	HI1001_LEGIII	HA1201_LEGIV	04-08-2010 00:37:30	1403	23-08-2011 10:02:30	150
OCC-PAL-051	5.896426	-162.12725	10.7	PRIA	Palmyra	STR	39368591566	HI1001_LEGIII	HA1201_LEGIV	04-08-2010 00:10:00	1406	24-07-2010 11:55:00	150
OCC-PAL-051	5.896454	-162.12723	10.17	PRIA	Palmyra	STR	39541654758		HI1001_LEGIII	16-09-2009 21:15:00	1236	04-08-2010 00:09:00	90
OCC-PAL-004	5.896679	-162.07818	5.5	PRIA	Palmyra	STR	5.69066E+11	HA1201_LEGIV		18-05-2012 01:19:59	1864	18-09-2012 20:28:59	60
OCC-PAL-004	5.89669	-162.07818	5.6	PRIA	Palmyra	STR	39301590872		HA1501_Line	19-09-2012 01:43:27	1890	16-04-2015 20:43:27	3600
OCC-PAL-004	5.896948	-162.07788	6.2	PRIA	Palmyra	STR	5604662		HA1801_Line	20-05-2016 19:49:59	2303	25-04-2018 08:44:59	300
OCC-PAL-004	5.896953	-162.07788	5.8	PRIA	Palmyra	STR	5.69066E+11	HA1501_Line		16-04-2015 21:34:59	2235	19-05-2016 01:49:59	300
OCC-PAL-053	5.897099	-162.12831	21.2	PRIA	Palmyra	SBE37	37SM265201887	HI1001_LEGIII	HA1201_LEGIV	14-04-2010 14:40:01	1401	16-05-2011 23:47:31	150
OCC-PAL-053	5.897103	-162.12822	20.61	PRIA	Palmyra	STR	39541654759		HI1001_LEGIII	16-09-2009 21:24:00	1235	04-07-2010 23:22:30	90
OCC-PAL-028	5.89713	-162.07829	10.1	PRIA	Palmyra	STR	5.69066E+11	HA1201_LEGIV		17-05-2012 23:06:59	1863	18-09-2012 00:33:59	60
OCC-PAL-003	5.897288	-162.07834	14.6	PRIA	Palmyra	STR	5.69066E+11	HA1201_LEGIV		17-05-2012 23:36:59	1862	18-09-2012 20:01:59	60
OCC-PAL-003	5.897302	-162.07828	14.5	PRIA	Palmyra	STR	39390381724		HA1501_Line	19-09-2012 04:45:27	1891	16-10-2014 16:45:27	3600
OCC-PAL-003	5.89733	-162.07817	14.3	PRIA	Palmyra	STR	5.69066E+11	HA1501_Line		17-04-2015 03:59:59	2233	17-05-2016 20:44:59	300
OCC-PAL-003	5.897341	-162.07817	14.4	PRIA	Palmyra	STR	5604663		HA1801_Line	20-05-2016 19:29:59	2302	08-05-2018 11:04:59	300
OCC-PAL-029	5.897374	-162.07834	19.1	PRIA	Palmyra	STR	5.69066E+11	HA1201_LEGIV		17-05-2012 22:50:59	1861	18-09-2012 11:23:59	60
OCC-PAL-002	5.897421	-162.07834	25.6	PRIA	Palmyra	STR	5604644	HA1501_Line		17-04-2015 07:04:59	2234	18-05-2016 14:59:59	300
OCC-PAL-002	5.897442	-162.07833	25.9	PRIA	Palmyra	STR	5604659		HA1801_Line	19-05-2016 23:59:59	2301	08-05-2018 02:54:59	300
OCC-PAL-002	5.897455	-162.07834	25.5	PRIA	Palmyra	STR	5.69066E+11	HA1201_LEGIV		17-05-2012 20:15:59	1860	14-06-2012 09:58:59	60
OCC-PAL-001	5.897525	-162.07834	29.6	PRIA	Palmyra	STR	39368591563	HA1201_LEGIV		19-05-2012 12:00:00	1867	18-09-2012 22:34:00	60

Table S7. Metadata for the temperature loggers used to quantify temporal patterns of cold pulses at the four corners of Palmyra's outer reef slope. Cold pulses were calculated using two loggers, one deep and one shallow, and are shown here as the pulse estimate at the deepest depth and the corresponding logger metadata used to derive that estimate. Additional information for each logger can be found in table S6 by looking up the Logger ID.

Site	Cold Pulse Depth	Start Year	End Year	Deep Logger ID	Shallow Logger ID	Deep Depth	Shallow Depth	Lat_deep	Lon_deep	Lat_shallow	Lon_shallow
East	25	2015	2017	2227	2228	25.7	15.2	5.876062	-162.001955	5.875603	-162.00379
East	25	2012	2015	1878	1877	26	14.8	5.876094	-162.001978	5.875648	-162.00391
North	15	2012	2014	1891	1890	14.5	5.6	5.897302	-162.078284	5.89669	-162.07818
North	15	2016	2018	2302	2303	14.4	6.2	5.897341	-162.078171	5.896948	-162.07788
North	15	2012	2012	1862	1864	14.6	5.5	5.897288	-162.07834	5.896679	-162.07818
North	15	2015	2016	2233	2235	14.3	5.8	5.89733	-162.078171	5.896953	-162.07788
North	15	2012	2014	1891	1890	14.5	5.6	5.897302	-162.078284	5.89669	-162.07818
North	15	2016	2018	2302	2303	14.4	6.2	5.897341	-162.078171	5.896948	-162.07788
North	15	2012	2012	1862	1864	14.6	5.5	5.897288	-162.07834	5.896679	-162.07818
North	15	2015	2016	2233	2235	14.3	5.8	5.89733	-162.078171	5.896953	-162.07788
North	25	2015	2016	2234	2235	25.6	5.8	5.897421	-162.078342	5.896953	-162.07788
North	25	2012	2012	1860	1864	25.5	5.5	5.897455	-162.078339	5.896679	-162.07818
North	25	2016	2018	2301	2303	25.9	6.2	5.897442	-162.078333	5.896948	-162.07788
North	25	2015	2016	2234	2235	25.6	5.8	5.897421	-162.078342	5.896953	-162.07788
North	25	2012	2012	1860	1864	25.5	5.5	5.897455	-162.078339	5.896679	-162.07818
North	25	2016	2018	2301	2303	25.9	6.2	5.897442	-162.078333	5.896948	-162.07788
South	15	2012	2012	1853	1851	14.6	5	5.869594	-162.07514	5.870108	-162.07509
South	15	2012	2014	1894	1895	14.7	5.5	5.869598	-162.075129	5.870118	-162.0751
South	15	2015	2016	2237	2238	14.2	4.9	5.869606	-162.075107	5.870139	-162.07512
South	15	2012	2012	1853	1851	14.6	5	5.869594	-162.07514	5.870108	-162.07509
South	15	2012	2014	1894	1895	14.7	5.5	5.869598	-162.075129	5.870118	-162.0751
South	15	2015	2016	2237	2238	14.2	4.9	5.869606	-162.075107	5.870139	-162.07512
South	25	2015	2016	2236	2238	24.4	4.9	5.869458	-162.075245	5.870139	-162.07512
South	25	2012	2014	1893	1895	24.53	5.5	5.869433	-162.075215	5.870118	-162.0751
South	25	2016	2018	2298	2299	24.4	14.2	5.869458	-162.075245	5.869736	-162.0755
South	25	2012	2012	1855	1851	25	5	5.869418	-162.07522	5.870108	-162.07509
South	25	2015	2016	2236	2238	24.4	4.9	5.869458	-162.075245	5.870139	-162.07512
South	25	2012	2014	1893	1895	24.53	5.5	5.869433	-162.075215	5.870118	-162.0751
South	25	2016	2018	2298	2299	24.4	14.2	5.869458	-162.075245	5.869736	-162.0755
South	25	2012	2012	1855	1851	25	5	5.869418	-162.07522	5.870108	-162.07509
West	25	2015	2018	2242	2240	24.7	14.5	5.88398	-162.168891	5.883225	-162.13321
